# Exploring New Algorithms for Molecular Vibrational Spectroscopy Using Physics-Informed Program Synthesis

**DOI:** 10.1021/acs.jctc.4c01312

**Published:** 2024-12-18

**Authors:** Kyle Acheson, Scott Habershon

**Affiliations:** Department of Chemistry, University of Warwick, Coventry CV4 7AL, U.K.

## Abstract

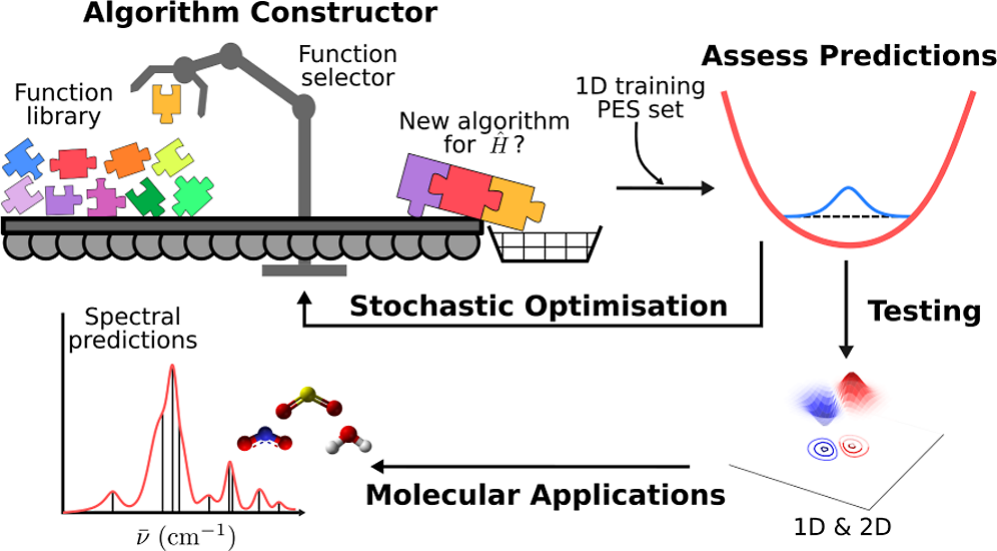

Inductive program synthesis (PS) has recently begun to emerge as a useful new approach to automatically generate algorithms in quantum chemistry, as demonstrated in recent applications to the vibrational Schrödinger equation for simple model systems with one or two degrees-of-freedom. Here, we report a new physics-informed approach to inductive PS that is more conducive to the generation of discrete variable representation algorithms for real molecular systems. The new framework ensures separability of the kinetic and potential operators and does not require an exact solution to compare synthesized algorithmic predictions with. Algorithms with a tridiagonal matrix structure are generated via a variational-based stochastic optimization procedure. Crucially, through an extensive testing procedure, we demonstrate that variationally synthesized algorithms perform just as well as those generated using a target function. Assuming a direct product representation of normal coordinates, these algorithms are applied to three triatomic molecules. In total, we identify a set of seven PS algorithms that accurately reproduce the vibrational spectra of H_2_O, NO_2_, and SO_2_, as predicted by Colbert–Miller and sine-DVR algorithms.

## 1 Introduction

The use of artificial intelligence and machine learning (AI/ML) in accelerating quantum chemical calculations and unraveling complex relationships in both experimental and theoretical observations is now well-established.^1−9^ Such methods typically involve training artificial neural networks (ANNs) or similar regression models to predict molecular properties given a set of input training data derived from experimental observations or quantum chemistry calculations. In the case of regression, one assumes a priori the underlying model structure, whereas ANN approaches involve implicitly transforming the data and optimizing a large number of floating-point weights to make predictions. The past decade or so has seen an explosion in computational and experimental chemistry analyses using such AI/ML strategies to predict and rationalize everything from identifying potential drug candidates^10^ to predicting crystal structures.^11^

An increasingly studied alternative strategy is symbolic regression^12−15^ (SR). SR attempts to find the best *mathematical expression* (i.e., function) that relates (or explains) the input data features to the observed (output) predictions, seeking to explore the expansive space of possible functions; in other words, SR provides a route to look “inside the black box” of more traditional AI/ML approaches^16^ and instead generate interpretive functions that more satisfactorily relate input/output relationships. Previously,^17,18^ we have shown how inductive program synthesis (PS), a subset of SR, can be used to automatically generate algorithms that yield solutions to the time-independent vibrational Schrödinger equation for bound potential energy surfaces (PESs). Importantly, in contrast to previous work in this domain,^4,19,20^ our approach does not yield algorithms that are specifically optimized to reproduce eigenstates and eigenvalues for a single PES only but instead generates algorithms that work equally well across a broad class of bound PESs (including PESs with more than one local minimum). The synthesized algorithms were shown to be similar to common discrete variable representation (DVR) algorithms by virtue of employing a uniform-grid representation of the PES and allowed eigenstates. Here, using a library of elementary functions and operations, operating in an iterative nested fashion on an initialized workspace matrix, algorithms are optimized stochastically by minimizing a target function that captures the difference between energy eigenvalues predicted by PS and those predicted by an oracle DVR algorithm. Importantly, energies are evaluated as expectation values over eigenvectors (wave functions) predicted by PS through diagonalization of the output workspace matrix. Much like the original DVR algorithms, such as the Colbert–Miller DVR (CM-DVR),^21^ these PS algorithms can be accurately extended to higher-dimensional model PESs, assuming an orthogonal coordinate system. Furthermore, it has been demonstrated that the PS algorithm search can be biased toward finding sparse-matrix approximations, providing significant computational speedup compared to standard DVR algorithms.

In this article, we follow these initial successes and explore modifications to our existing PS scheme that facilitate the generation of algorithms that can be accurately applied to molecular systems. Specifically, we introduce a new stochastic optimization procedure based on the variational principle, removing the previous requirement for numerically exact reference solutions when quantifying the predictions of PS algorithms. After using this updated strategy to generate a new set of PS-generated algorithms for vibrational wave function prediction, the best-performing PS algorithms are validated in simulations of three triatomic molecules (H_2_O, NO_2_, and SO_2_), and we demonstrate that they yield vibrational transition energies within 1 cm^–1^ of traditional DVR schemes. As such, these results, marking the first time that a computer-optimized algorithm has been used to study molecular vibrational spectra, demonstrate that PS may be a useful alternative to traditional AI/ML methods for accelerating quantum-chemical calculations.

## 2 Theory

The general PS procedure used here is similar to that employed in previous work.^17,18^ However, here we aim to synthesize DVR-like algorithms that can be directly applied to vibrational spectra prediction in molecular systems. Following a short review of the PS setup, we proceeded to discuss several improvements to the methodology that make this possible.

### 2.1 Problem Definition

We aim to synthesize algorithms capable of solving the time-independent vibrational Schrödinger equation
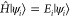
1to give vibrational eigenstates |ψ_*i*_⟩, from which the energies are calculated as an expectation value
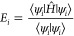
2As addressed in previous work,^18^ we choose to evaluate energies as an expectation value due to the degeneracy of the eigenvalue problem; any PS algorithm corresponding to a relatively broad class of matrix functions (e.g., those that can be written as Taylor expansions) can yield the same set of eigenstates as the original Hamiltonian matrix but with different eigenvalues. As such, we focus on using PS to generate *functions* of the Hamiltonian matrix with accurate eigenstates, noting that the corresponding eigenvalues can always be evaluated through expectation values.

Here, we define the Hamiltonian using a set of orthogonal coordinates **q**, for example, representing the normal-modes of the system of interest
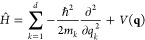
3This Hamiltonian is simply the sum of the kinetic energy operator (KEO) for each of the *k* degrees of freedom (DOFs) (each of which are associated with a mass *m*_*k*_) and the PES operator *V*(**q**).^22^ We note that as is typical in vibrational spectra calculations, we restrict ourselves to the electronic ground-state PES.

We focus on finding solutions of eq 1 in a DVR basis, which can generally be constructed by diagonalization of the position operator (*q̂*) in the basis of orthogonal polynomials.^23^ The eigenvalues of the matrix representation of *q̂* correspond to the abscissas associated with the underlying Gaussian quadrature. In this work, we compare the predictions of PS-generated algorithms to those calculated from both a DVR of sine functions and the generalized DVR developed by Colbert and Miller,^21^ both of which correspond to an underlying uniform grid of quadrature points.

**Figure 1:**
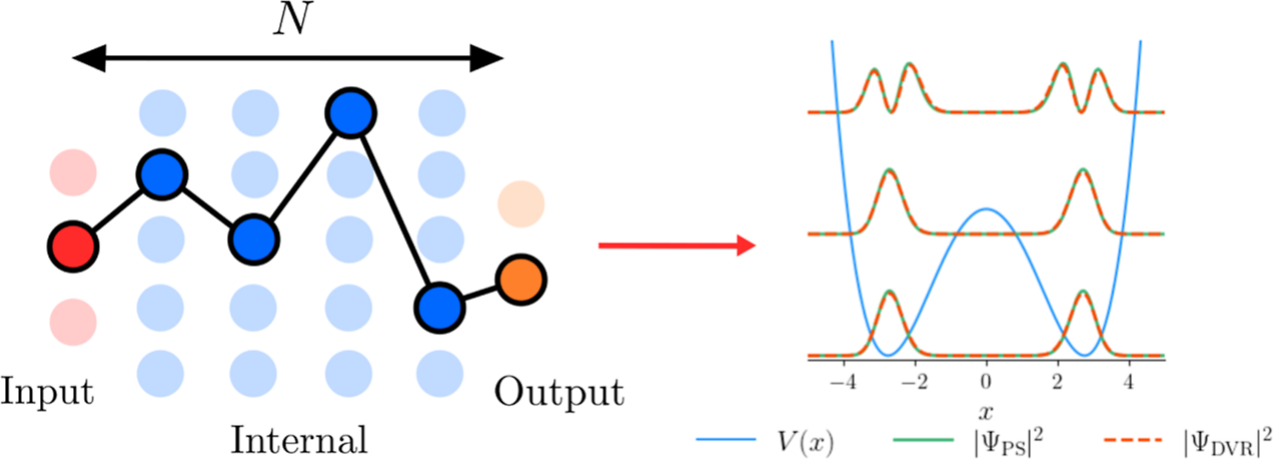
Program space represented as a network comprising a single input layer (red), *N* – 2 internal layers (blue), and a single output layer (orange). The total number of layers (*N*) corresponds to the number of elementary operations the program is constructed from. The darker circled nodes represent the selected functions from which the given program is constructed. On the right-hand side, the output wave function and energies are compared to those from a reference DVR algorithm, here CM-DVR.

As with our previous work,^17,18^ we aim to synthesize computationally advantageous algorithms that construct an approximate Hamiltonian-function matrix that yields vibrational wave functions and energies for a given 1D problem potential. In this work, we choose to focus solely on the generation of algorithms that use tridiagonal matrices, providing significant savings in both computer time and memory. This is achieved through stochastic optimization of linearly structured programs comprising *N* total elementary operations selected from a larger function library, each of which performs some operation on a workspace matrix (**M**) that approximates the Hamiltonian (or a function of the Hamiltonian, as described below). This concept is demonstrated in Figure 1, representing a program comprising a set of input, internal, and output functions. The input layer serves to initialize the workspace matrix **M**, for example, as the identity matrix, whereas the internal layers perform a series of further mathematical operations on this matrix, and, finally, the output layer yields a series of vectors that aim to approximate the allowed eigenstates of the system. At each layer, the number of nodes corresponds to the total number of available functions, with the internal structure containing *N* – 2 independent layers. A given program can therefore be thought of as a path through this network, which can be subsequently assessed based on how accurately it reproduces the vibrational eigenstates and energies calculated using an oracle DVR algorithm, as seen in the right of the figure.

### 2.2 Improvements to PS

Having reviewed the general problem definition and previous applications of PS to vibrational eigenstate prediction, we now highlight several improvements to our original PS setup, which aim to facilitate the discovery of more efficient algorithms that can be readily applied to higher-dimensional molecular systems.

#### 2.2.1 New Problem Representation

In our previous work, we demonstrated that the synthesized algorithms generate a workspace matrix that approximates a function of the Hamiltonian for a given set of randomly generated 1D bound-polynomial PESs.^17,18^ In training, the selected functions operated on the total workspace matrix, meaning that the kinetic-energy and potential-energy matrix elements are not always guaranteed to be separable as they are in typical DVR algorithms.^22^ In this article, to ensure separability of the kinetic-energy and potential-energy matrices—thereby potentially simplifying algorithms structure—we assume that the potential-energy matrix is diagonal in the underlying (yet unknown) basis and is simply given by the value of the potential at each grid point.^24^ As such, the internal function set now includes no operations that involve the potential energy operator, and so all input and internal functions that act on the workspace matrix **M***only* now aim to approximate the action of the KEO. At the output layer, the potential is added to the diagonal of the workspace matrix, **M**, yielding the final matrix that is diagonalized to approximate the system eigenstates represented on the underlying coordinate grid. As such, the function library is now composed of 85 total functions (4 input, 80 internal, 1 output); these are given in full in the Supporting Information. As discussed below, an important benefit of this approach is that one can more easily expand the PS-generated expressions for matrix elements into more than one dimension. Furthermore, as we discuss later, we find that we are able to generate high-performing programs using significantly fewer operations than previously reported.^17,18^

Reflecting the focus of the current article on *vibrational spectroscopy* of bound molecular systems, each independent PS run used a set of 1D training PESs drawn from one of five basic PES types. These include harmonic and anharmonic oscillators, Morse potentials, and both symmetric and asymmetric double wells. For each of these types, a training set of *N*_train_ potentials is generated with a range of force constants, dissociation energies, equilibrium positions, and barrier heights. To facilitate the discovery of algorithms that work over a general number of grid points *N*_g_, the training set contains potentials defined by randomly selected values of *N*_g_. Unlike in our previous work where we generated random 1D polynomial PESs,^17,18^ the potentials that form the training set here are carefully selected to tackle a wider variety of real-world molecular vibrational problems.

#### 2.2.2 Pseudovariational Code Evaluation

An important challenge in our previous applications of PS was the requirement of having available a so-called oracle code that could yield the numerically exact eigenstates and eigenvalues for generic 1D PESs; these exact results can then be used as “targets” for PS optimization. In our previous studies, we employed the well-known CM-DVR; of course, it would be more beneficial to remove this requirement in the first place such that PS optimization and validation did not rely so explicitly on an existing methodology.

Here, we introduce a new optimization approach based on the variational principle that does not require the use of an oracle DVR code for comparison of eigenvalues. We will compare the results of this new approach to that introduced previously,^18^ which is based on minimization of the target function defined as
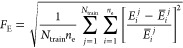
4Here,  is the energy eigenvalue given by an oracle DVR algorithm for the *i*th eigenstate of the *j*th problem potential in the training set. The energy eigenvalue *E*_*i*_^*j*^ is predicted directly from the output eigenstates given by PS, and it is calculated from the energy expectation value, as in eq 2. However, evaluating the energies using eq 2 requires a continuous form of the wave function in evaluating the contribution from the KEO due to the presence of the second derivative term. Previously, we used kernel ridge regression to generate a continuous form of the wave function. We find that using a constant kernel width is not always guaranteed to yield an accurate representation of the wave function and may require optimization in cases where an excessive number of grid points *N*_g_ outside the range of original training data set is used. To overcome this, we take the approach of evaluating the KEO contribution in momentum space, which assuming atomic units is given by
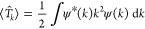
5where *k* is the wave vector. This gets rid of the requirement for a continuous wave function due to the lack of second derivatives, making the energy evaluation much easier, provided one can compute the Fourier transform of the real-space wave function. We note that the potential expectation value is still evaluated as
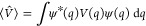
6Following the generation of the training set of *N*_train_ PES examples, an initial program comprising *N* functions randomly selected from the function library is generated. This program is represented by a state vector **S** of length *N*, comprising a series of integer function IDs. Optimization of a PS program then proceeds via simulated annealing (SA), which involves minimizing the target function in eq 4, starting at the initial effective temperature of *T*_init_. At each of the *N*_iter_ iterations, the current state of program defined by **S** is perturbed by randomly changing a number of function IDs. The new perturbed algorithm **S**_new_ is then accepted or rejected based on the Metropolis criterion, where the acceptance probability is given by
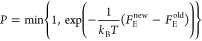
7with *T* the current effective temperature, and *F*_E_^new^ and *F*_E_^old^ are the values of the target function for program states **S**_new_ and **S**, respectively. Over each of the iterations, the current temperature is reduced according to a linear cooling schedule.

Having reviewed the existing optimization strategy and energy evaluation using PS algorithms, we now highlight the new optimization procedure employed in the PS simulations reported below. Given that the energies defined by eq 2 obey the variational principle,^22^ we consider the following question: can one directly minimize an energy functional to find optimal wave functions using PS? This would remove the need to train PS algorithms using a target function that requires exact reference solutions as in eq 4. However, there are a few important caveats that must be addressed. First, given that we aim to synthesize algorithms that are DVR-like, these algorithms may not always be guaranteed to provide an upper limit on the energy. It is well-known that DVR algorithms may yield energies lower than the variational limit at a small number of grid points and may converge to the correct energy “bottom-up” as the number of points are increased.^24^ This results from the fact that in addition to the error from a finite basis truncation, in the DVR representation, one incurs an additional quadrature error, which can also be thought of as a nonzero first-order perturbation correction.^25^ For this reason, we refer to our optimization as being pseudovariational, and any algorithm that is trained on a small number of grid points must also be shown to converge in the large grid-size limit. Second, this approach formally limits us to only minimizing the energy of ground vibrational states, potentially meaning that algorithms synthesized using this approach may not give accurate energy predictions for higher eigenstates. However, as we shall discuss in the Results section, it is generally possible to find algorithms that also give accurate energy predictions for higher eigenstates; we hypothesize that this is a direct consequence of the fact that the eigenstates obtained by PS are naturally orthogonalized, so optimization of the PS-generated ground-state vibrational eigenstates imposes constraints on the higher-energy eigenstates.

With these caveats in mind, we define the energy vector **E** of length *N*_train_, with elements defined as the ground-state energy expectation value predicted by PS
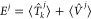
8Here, the index *j* runs over all PESs in the training set. In addition, the operator expectation values are defined for the given problem *j* as in eqs 5 and 6. In the pseudovariational optimization approach, we replace the target function previously given in eq 4 with the energy *vector* in eq 8. Note that the optimization still proceeds via SA, with algorithms updated using the Metropolis criterion. In addition to Metropolis acceptance, the algorithm update criteria for the current best algorithm are now also given by the following two conditions:

1. For the perturbed algorithm in state **S**_new_ (with energy vector **E**_new_) and the current best algorithm **S** (with energy vector **E**), the new algorithm is accepted only if there exists at least one training-problem *x* for which *E*_new_^*x*^ < *E*^*x*^.
2. For all other problems *j* (where *j* ≠ *x*), the elements of the energy vector **E**_new_ must obey the inequality *E*_new_^*j*^ ≤ 1.005 × *E*^*j*^. In other words, the ground-state energies of all problems do not increase by more than 0.5% relative to the previous best PS estimate.

The benefit of this conditional approach is that it accounts for the fact that different problems may converge at different rates and, provided that a perturbation to an algorithm always lowers at least one of the problem energies and does not increase the others, the algorithm will always be further improved. Moreover, due to the stochastic nature of the optimization, it is necessary to provide some flexibility in the second condition above by allowing minor increases in the energies of the other problems. Finally, we note that the imposition of these conditions, in addition to the Metropolis criterion, means that detailed balance is no longer realized, but this is not relevant in the current application of SA as an optimizer.

Since we no longer have a target function to identify the best synthesized algorithms with reference to an oracle solution, we must finally define a new method of assessing the overall algorithm performance across multiple target problems. For this, we simply sum the elements of the energy vector of each given PS run to give a single-value measure *E*_run_; the value of *E*_run_^min^ can then be used as a simple comparison between different PS runs. For a large set of PS-generated algorithms, any algorithms that have a value of *E*_run_ ≤ 1.01 × *E*_run_^min^, are typically selected for further analysis and testing, as explored below.

### 2.3 Molecular Spectroscopy Calculations

Following training and testing of PS-generated algorithms obtained via the two optimization methods discussed above, we will subsequently use these algorithms to calculate the vibrational transition energies for molecular systems; these simulations will mark the first time that computer-discovered algorithms have been applied to study “real-world” molecular systems. Here, we outline the multidimensional DVR approach used to calculate molecular vibrational eigenfunctions and also give details of the molecules studied and PESs employed.

#### 2.3.1 Multidimensional DVR

In an orthogonal coordinate system, such as that defined in eq 3, the Hamiltonian matrix elements can be easily extended to higher dimensions as a result of the separability of a KEO with no mixed second derivatives. For example, in a system with two DOFs (*q*_1_, *q*_2_), Hamiltonian matrix elements can be written as^24^
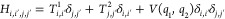
9Here, (*i*, *i*′) are the indices of grid-points along *q*_1_, (*j*, *j*′) similarly being the indices of grid-points along *q*_2_, and  is an element of the one-dimensional KEO matrices. Note that here the total KEO contribution can be simply evaluated using the two one-dimensional representations of the KEO for each DOF. More generally, a system with *d* DOFs defined on a direct product grid of *n* grid points along each DOF results in a rank-2*d* Hamiltonian of size *N*_g_ × *N*_g_, where the total number of grid-points is *N*_g_ = *n*^*d*^. Thus, a tetra-atomic system defined by *n* = 20 grid points results in *N*_g_ = 6.4 × 10^7^, yielding a Hamiltonian with 4.096 × 10^15^ total matrix elements. For this reason, one might consider the use of alternative discretization schemes that do not involve a direct-product grid. For example, in nondirect-product grids, one can develop schemes employing an energy cutoff to prune grid-points that sample high-energy regions of the PES where the wave function amplitude would be expected to be negligible.^24^ Such approaches have been employed in a variety of truncation and contraction schemes;^24,26−30^ however, they are often not generally applicable without prior insight into the structure of the PES. The advantage of the standard direct-product representation in eq 9 lies in its generality and ease of evaluation of the matrix elements, albeit at the expense of large matrix sizes. In this article, we aim to compare PS algorithms that are generally applicable, much like CM-DVR^21^. Thus, all PS algorithms trained in one-dimension can be readily extended to higher dimensions, assuming a direct product of normal-mode coordinates.

While the DVR Hamiltonian matrix is typically sparse, it can still quickly reach sizes that are too large to store in computer memory, even in compressed sparse storage formats. As a result, for systems with more than three DOFs, we will generally turn to iterative matrix-vector product eigensolvers such as those based on the Lanczos algorithm.^24,30,31^ Here, speed-up can be achieved by directly exploiting the structure of the Hamiltonian, in addition to matrix sparsity. This approach involves factorizing the *d*-dimensional Hamiltonian operator into a product of 1D operators. For example, the matrix representation of the *d*-dimensional KEO can be written as the sum of products of *d* 1D KE matrices. To illustrate, let us denote **T**^**k**^ as the *n* × *n* KE matrix for DOF *k*. To calculate the product **Tv**, for each *n* × *n* matrix **T**^**k**^, we compute its product with *n*^*d*–1^ subvectors each of length *n*, which has a time complexity of *n*^*d*+1^. This involves treating the vector **v** as a tensor; for each of the matrices **T**^**k**^, a permutation of indices is performed and the tensor is transformed to a matrix of shape *n* × *n*^*d*–1^, which is then matrix-multiplied with **T**^**k**^. In total, there are *d* 1D matrices **T**^**k**^ for which this process must be repeated. Thus, the total complexity is *d*(*n*^*d*+1^), which is significantly lower than the *n*^2*d*^ multiplications required in the full matrix representation (we note that we assume no sparse matrix storage formats in this simple analysis). Further information on this procedure can be found in the Supporting Information.

We note that we have so far only considered the case where the matrices **T**^**k**^ have nonzero matrix elements. As shown below, PS can be employed to generate PS algorithms that have a tridiagonal KEO matrix structure by construction; as such, further speed-up can be obtained in the multiplication of each **T**^**k**^ matrix with the *n*^*d*–1^ subvectors of **v**. As a final aside, we note that the product of the diagonal potential matrix with the vector **v** can trivially be calculated and subsequently added to the result.

#### 2.3.2 Molecular Applications

To ensure accurate calculation of the vibrational transition energies, one not only requires enough grid points in the discretization of nuclear vibrational wave functions but also an accurate PES.^24^ Sampling a sufficiently large coordinate range of the PES configuration space for the energy range of interest can incur significant computational cost. Taking a general tetra-atomic system with 3*N* – 6 = 6 DOFs as an example, assuming a direct product grid of *n* = 20 points along each DOF, this would require 6.4 × 10^7^ evaluations of the PES. Performing such a large number of ab initio calculations at an accurate level of electronic structure theory (such as multireference configuration interaction, or coupled-cluster) quickly becomes infeasible. For this reason, a common approach is to instead generate a “spectroscopic” PES—in other words, a reduced PES that is generated using a combination high-level ab initio data and experimental observations.^32−42^ Given an accurate spectroscopic potential, it then becomes possible to quickly perform many evaluations of the PES for a range of coordinates.

In this work, we demonstrate the performance of PS-generated algorithms (that are found to converge on accurate results for model two-dimensional problems, as discussed below) for a set of three triatomic molecules, namely, H_2_O, NO_2_, and SO_2_. In the case of H_2_O, we use the well-known Partridge–Schwenke^32^ model to describe the (Born–Oppenheimer ground-state) PES. However, without access to accurate spectroscopic PESs for NO_2_ and SO_2_, we instead directly generate PESs using DFT calculations; in both cases, we employ the B3LYP/def2-TZVP level of theory. We note that, while this DFT calculation setup is generally expected to be less accurate than high-level spectroscopic PESs, the focus of this work is to demonstrate that both our PS-generated algorithms and known DVR algorithms converge to the same predictions for vibrational eigenstates while also being faithful reproductions of experimental observables.

For each triatomic molecule studied here, we use a direct product grid that includes 41 grid points for the lowest frequency bending mode and 31 points for both symmetric and asymmetric stretches, amounting to a total of 39,401 DFT calculations. While this is feasible in three-dimensional systems at the DFT level-of-theory, one quickly runs into sampling problems when studying higher dimensionality or using more accurate electronic structure approaches.

To address this challenge, we turn to an alternative approach to the problem of sampling configuration space when fitting PESs for calculations of vibrational spectra, particularly focusing on employing nonproduct coordinate grids. In recent years, the use of nonproduct Smolyak grids and quadrature rules for both representing the potential and evaluating matrix elements has received considerable interest within the vibrational spectroscopy community.^43−49^ However, herein, we investigate the use of Sobol sequences^50^ in sampling points for PES generation. Sobol sequences have been widely applied to a variety of sampling problems, with the most relevant example for this work being the sampling around the time-dependent wave packet generated in an on-the-fly approach to the multiconfigurational time-dependent Hartree (MCTDH) method.^5,51,52^ Sobol sampling employs a quasi-random generation of sequences such that the discrepancy between points is minimized; further details on the procedure can be found in the Supporting Information. In short, this allows for the generation of coordinate points that maximally span the sampling space while minimizing overlap between points. Note that an important caveat to this approach is that the number of sampling points should generally be a power of base-two to ensure the balance properties of Sobol sequences.^50^ As such, we generate a three-dimensional Sobol sequence consisting of just 128 configurations bounded by each target molecule’s normal-mode coordinate limits. The DFT-calculated PES values at each of these points then serve as the training set for building a Gaussian process regression (GPR) model using standard radial basis functions. Following the identification of the kernel’s optimal length-scale by maximization of the log-marginal likelihood, the GPR model was subsequently used to predict the value of the PES on the full direct-product grid of 39,401 points, thereby minimizing the number of DFT calculations required in this application. We note that, while use of GPR in accurately predicting PESs is well-known,^1,3,8,9^ the focus here lies on its combination with Sobol sampling in accurately reproducing vibrational transitions energies calculated via DVR.

All calculations of vibrational transition energies are performed using our own publicly available software package, which iteratively finds the eigenvalues and eigenvectors of the Hamiltonian, as described in Section 2.3.1. This is achieved by using the implicitly restarted Lanczos method^31,53^ to find the *n*_e_ lowest eigenvectors. We note that, in the case of PS-generated algorithms, the energies are re-evaluated as the expectation value of each of the *n*_e_ eigenvectors, as described previously.^17,18^ This employs a *d*-dimensional fast Fourier transform of the eigenvectors into momentum space in order to calculate the KE contribution, as shown in eq 5. The vibrational transition energies obtained from PS-generated algorithms for each molecule are finally compared to values calculated using both CM-DVR and sine-DVR approaches.

## 3 Results and Discussion

Here, we begin by discussing the impact of our updated PS specification in generating DVR-type algorithms that can be applied to molecular systems. We also compare the results of our existing optimization approach, based on eq 4, to the new pseudovariational optimization, which does not require an oracle solution for training. Next, we discuss the extension of our PS-generated algorithms to many-dimensional systems, including a discussion of a more rigorous PS testing strategy than previously employed. Finally, we conclude with a comparison of our PS-generated algorithms to standard CM-DVR and sine-DVR calculations of vibrational spectra for H_2_O, NO_2_, and SO_2_.

### 3.1 PS Optimization Details

Here, we summarize the input parameters used to synthesize new algorithms via SA optimization. In the initial training step, we use *N*_train_ = 20 1D PESs, comprising five of each class of PES from sets of harmonic oscillators, Morse potentials, symmetric double wells, and asymmetric double wells. This variety of PESs were generated using a range of grid-sizes *N*_g_ ∈ [31, 251] to ensure that the synthesized algorithms can be applied to any grid-size. The total number of independent SA optimization runs was *N*_run_ = 200, each of which ran for a total of *N*_iter_ = 5000 SA iterations. For each SA run, the initial temperature was *T*_init_ = 5 × 10^3^ K. With the PS representation employed here, in which we add the potential to the diagonal elements of the workspace matrix **M**, we find that accurate algorithms can be generated with as few as *N* = 10 total operations. This corresponds to one operation from the input layer, eight from the internal layer, and one from the output (as demonstrated in Figure 1). Furthermore, here we only focus on generating algorithms that use a tridiagonal workspace matrix; as a result, each operation selected from the function library only acts on the tridiagonal matrix elements.

In the standard optimization approach, which involves minimization of eq 4, the PS-generated algorithms were trained on the first *n*_e_ = 3 eigenstates for each PES. In this case, the exact (target) energies used in the target function were calculated using sine-DVR with a dense grid of *N*_g_ = 501 points. For the pseudovariational optimization, which minimizes the energy vector **E** of the 20 target PESs, only the lowest-energy eigenstate is included as an optimization target. For the set of best algorithms, we subsequently test that the energies of all three eigenstates converge in the large grid limit at *N*_g_ = 501 points. Those PS-generated algorithms that converge satisfactorily are then subjected to further testing in higher-dimensional systems, as described below.

### 3.2 Comparison of Optimizations

**Figure 2:**
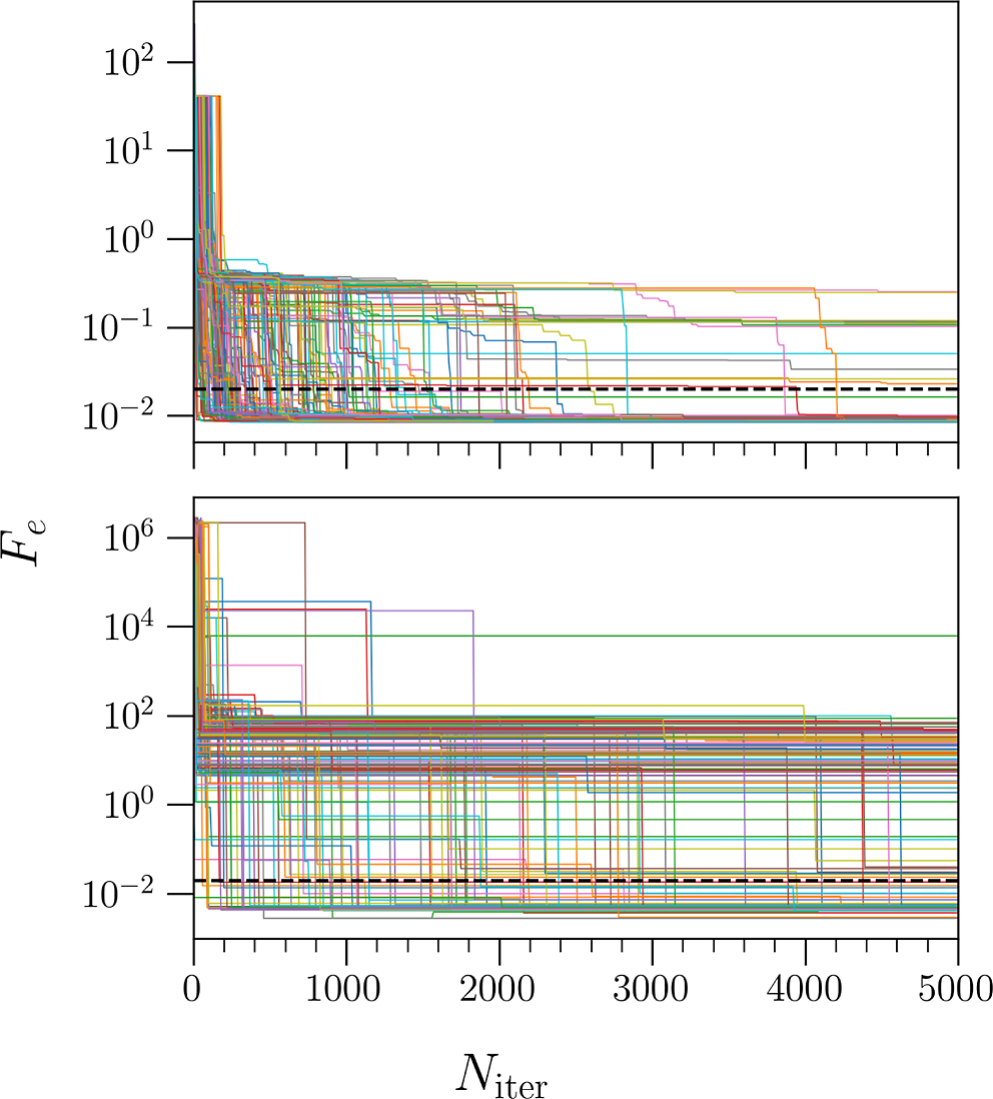
Value of the target function *F*_e_ as a function of iteration number *N*_iter_ for 200 runs of PS using the target function (top panel) and using the problem energy vector in the variational optimization (bottom panel). Values in the top panel include a sum over *n*_e_ = 3 eigenstates in eq 4, whereas those in the bottom panel include only the ground state. The black dashed line represents the threshold of 2 × 10^–2^. Note, both panels are plotted on a logarithmic scale.

As an initial comparison of the two optimization methods studied here, Figure 2 shows the value of the optimization function *F*_e_ over each iteration for all 200 SA runs. Here, the upper panel shows the runs that use the target function in the optimization, whereas the lower panel shows pseudovariational optimization. Note that while the latter does not depend on the target function, we simply plot its value for comparative purposes. In each panel, we see that a number of runs converge below the threshold of *F*_e_ = 2 × 10^–2^, indicated by the dashed black line. In the top panel, 183 of these runs converge below the threshold, with most of them converging below 2000 iterations. Comparatively, for the pseudovariational optimization, 80 algorithms converge below the threshold. While this is a significantly smaller number, it is still significant. Here, we also see that the number of iterations for convergence generally spans a wider range of values, with more runs converging after around 2000 iterations.

**Figure 3:**
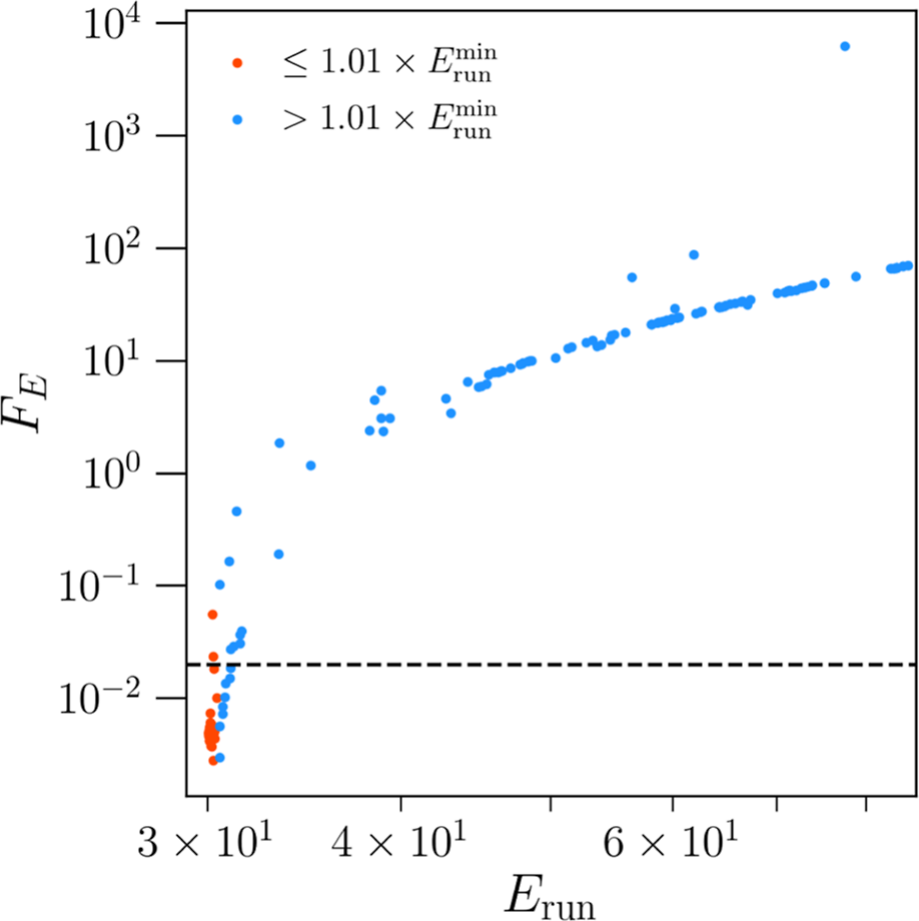
Value of the target function (*F*_E_) vs the value of the summed energy vector (*E*_run_) for each of the 200 runs obtained using variational optimization. Orange points correspond to runs that are within 1% of the minimum summed energy (*E*_run_^min^) of all runs. This measure reflects the best optimized algorithms, as selected according to the target function threshold of 2 × 10^–2^ indicated by the dashed black line. Note, both axes are plotted on a logarithmic scale.

In the case of the target function optimization, we select all algorithms under the threshold of 2 × 10^–2^ for further tests. While we show the value of target function for the pseudovariational optimization in Figure 2, this information would not be available in the general case when the numerically exact results are not available. Therefore, as discussed in Section 3, we propose to select algorithms that yield a summed energy vector *E*_run_ within 1% of the minimum value of all runs *E*_run_^min^. Turning to Figure 3, we show each run’s value of *E*_run_ plotted against its corresponding target function value. The dashed line across the *y*-axis at *F*_E_ = 2 × 10^–2^ again represents the target function threshold. Generally, we see that those runs with a lower value of *E*_run_ also exhibit low target function values. However, this relationship is not linear, and the value of *F*_E_ increases exponentially with *E*_run_. We then proceeded to select all algorithms that are within 1% of *E*_run_^min^ for further testing. This is highlighted by the 73 runs that are represented by orange-colored data points in the figure.

### 3.3 Identifying Transferable Algorithms

From the simulations above, it is clear that both optimization strategies yield a number of algorithms that exhibit a good level of performance when they are evaluated for the training set of PESs. The next challenge is to further prune these PS-generated algorithms to identify the best-performing, yet generalizable, algorithms for applications to molecular problems.

#### 3.3.1 Testing in One-Dimension

While both optimization methods appear to be capable of finding algorithms that can accurately predict energies of the 20 PESs that make up the training set, several additional criteria must first be met before any PS-generated algorithm is considered further.

**Figure 4:**
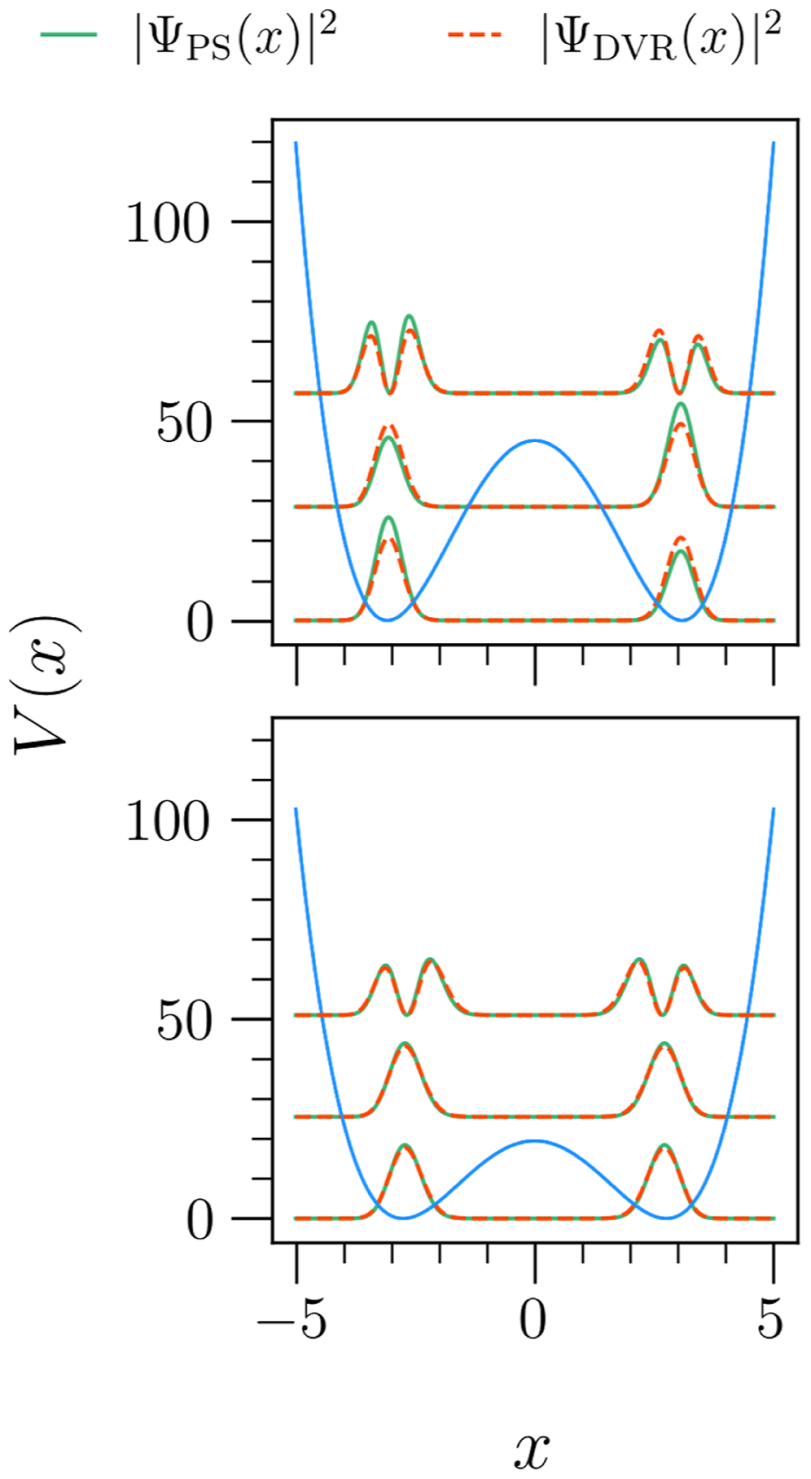
Probability amplitudes of the first three wave functions predicted by a PS algorithm and the oracle DVR code for a symmetric double-well with a large energy barrier (upper panel) and one with a smaller barrier (lower panel). In the upper panel, we see that this particular PS-generated algorithm predicts unequal amplitude in each PES well, whereas this is not seen when the barrier is smaller (lower panel). Despite this, the PS-predicted eigenvalues for both double-well PESs are within a fractional error Δ*E*_F_ = 0.005 for all three lowest eigenstates. Note, the probability densities of the eigenstates are not plotted at their eigenvalues and are instead offset by a constant factor for visibility. However, the first even/odd pair of eigenstates is predicted to be degenerate.

First, while a large number of synthesized algorithms predict accurate energies for the training data set, we found that, for *symmetric* double-well potentials, some algorithms unexpectedly predicted wave functions with unequal amplitude in each minima. Crucially, for the majority of double well potentials within the training set, this was not the case and only applied to potentials with a very high energetic barrier. This can be seen in the top panel of Figure 4, where the PS-predicted probability amplitude in each well for the first three eigenstates is not equal. Turning to the lower panel, representing a PES with a lower energetic barrier, the same algorithm predicts equal amplitude, more closely resembling the oracle DVR algorithm. Interestingly, all fractional errors for the first three eigenstates, calculated as , are less than 0.005 (0.5%). Given that for a pair of odd/even eigenstates, the correct probability density is known to be close to that of a single Gaussian expansion in one of the wells, it is perhaps not surprising that some PS algorithms observe symmetry breaking for high tunneling barriers. However, we exclude any PS-generated algorithms that predicted such nonsymmetric wave functions from further testing. Furthermore, we note that the PS algorithms are assessed on their eigenvalue predictions and not tunneling splittings directly. As accurately capturing small tunneling splittings is a separate and difficult computational challenge in itself, we leave this for future work.

As a second test to further prune the PS-generated algorithm set, we subsequently confirm that the energies of each algorithm correctly converge in the limit of a large grid-size; as a reminder, we intentionally optimize algorithm performance for a range of grid-sizes but do not explicitly include fully converged grid-sizes for a given problems. As such, this additional test ensures correct convergence behavior with grid-size, as an alternative to imposing this behavior during optimization.

**Figure 5:**
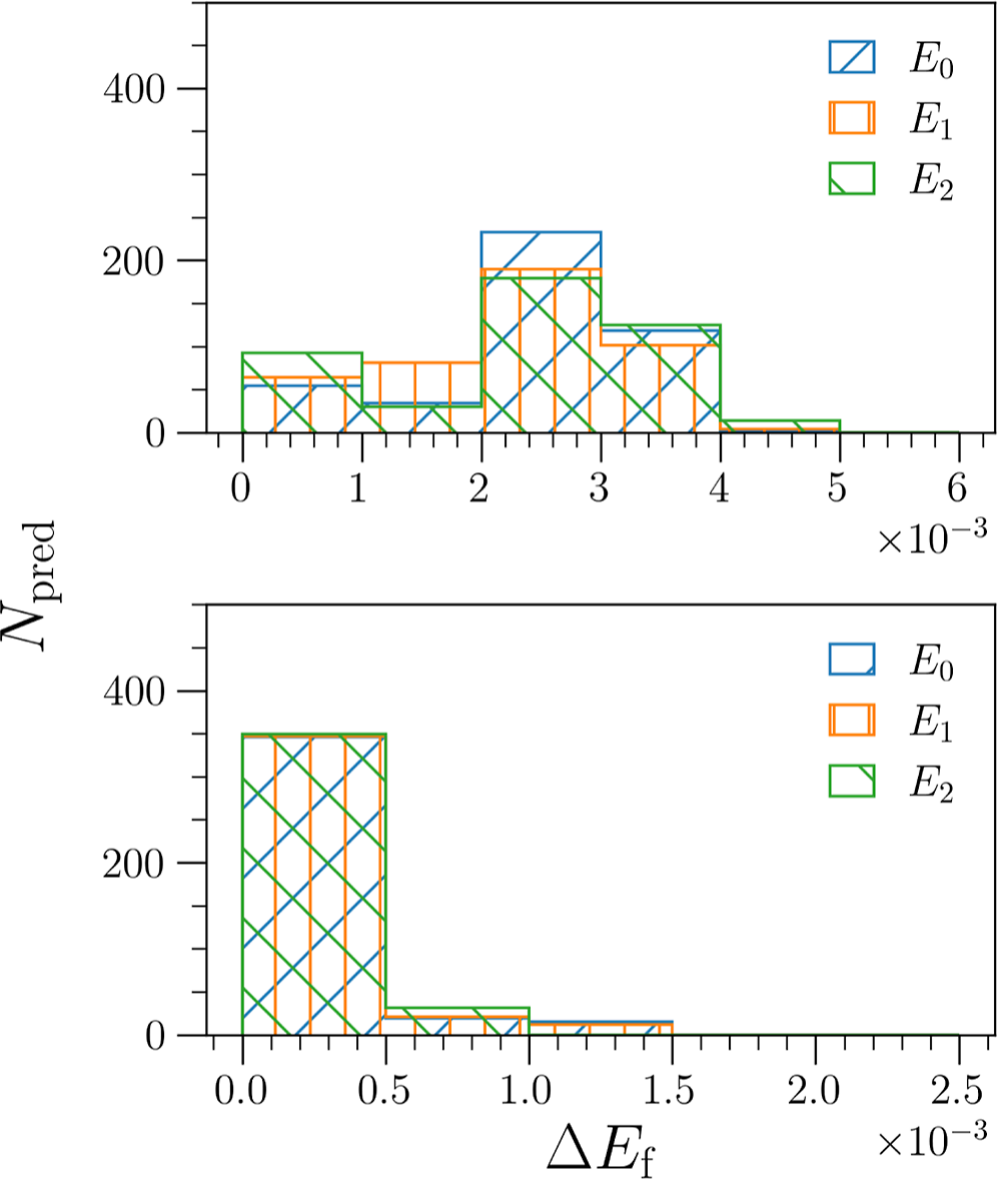
Histogram of fraction errors Δ*E*_F_^*j*^ in the large grid limit (*N*_g_ = 501) of the 20 problem potentials in the training set. The fractional errors of each of the three eigenstates are plotted individually. Note that Δ*E*_F_^*j*^ ≤ 0.005 (0.5%) for all predictions, with the pseudo-variational algorithms (bottom panel) exhibiting even lower errors that are more narrowly distributed.

To check the convergence behavior of PS-generated algorithms, we again use the fractional energy error, now calculated with reference to the energies predicted by the oracle DVR algorithm in the large grid-size limit of *N*_g_ = 501. In the case of algorithms generated through target function optimization, we find that only 22 of the initially optimized algorithms converge to within a fractional energy error of Δ*E*_F_^*j*^ = 0.005 (0.5%) at *N*_g_ = 501. In contrast, for the pseudovariational optimization, we find that 19 algorithms converge to this target fractional energy error. This can be seen in Figure 5, where we see that despite being trained on only one eigenstate, the pseudovariational algorithms (bottom) observe lower and more narrowly distributed errors than the target-function trained algorithms (top). These results demonstrate that it is possible to synthesize novel algorithms that guarantee energy convergence when only trained on a range of small grid-sizes and also serve to highlight that the method of optimization has little bearing on the final convergence performance.

Following this check of PS-generated algorithm convergence in the large-grid limit with respect to the training set, we then proceeded to test the transferability of the algorithms to other PESs. This involved the random generation of *N*_test_ = 500 bound-polynomial PESs according to the same procedure employed in previous work.^18^ To check the convergence of the predicted energies for the first *n*_e_ = 3 eigenstates, each individual potential is generated at nine different grid sizes *N*_g_ ∈ [31, 501].

**Figure 6:**
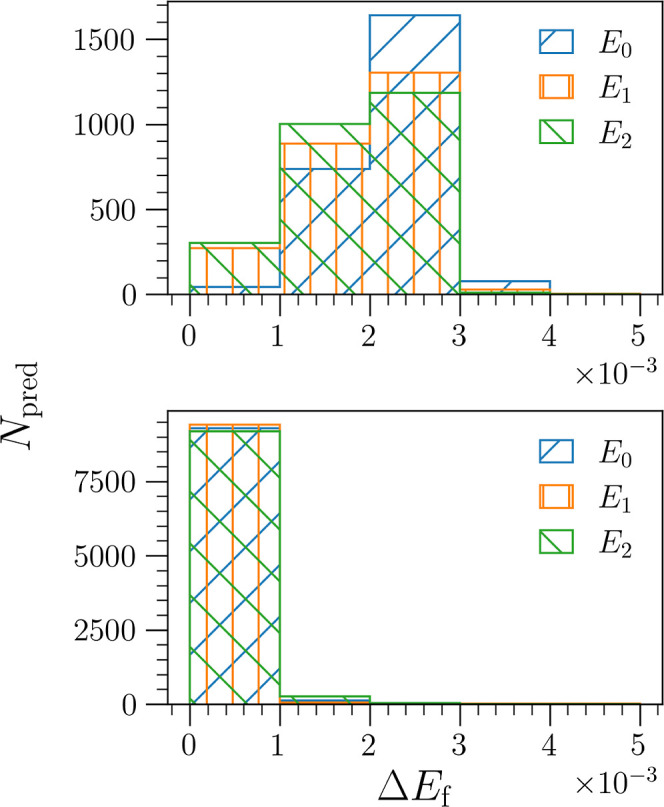
Histogram of fractional errors Δ*E*_F_^*j*^ in the large grid limit (*N*_g_ = 501) of the 500 problem potentials in the testing set. The fractional errors of each of the three eigenstates are plotted individually. Note that Δ*E*_F_^*j*^ ≤ 0.005 (0.5%) for >99% of predictions, with the pseudo-variational algorithms (bottom panel) again exhibiting even lower errors that are more narrowly distributed.

Here, a PS algorithm is categorized as converged with respect to the test set of 500 polynomial potentials if the predicted energies of all three eigenstates are within a fractional error of Δ*E*_F_^*j*^ ≤ 0.005, when evaluated in the large grid limit. According to this criterion, we find a set of five target-function trained PS algorithms converge with respect to the test set; we label this set **A**. In the case of PS algorithms obtained by variational optimization, we define the corresponding set **B**, comprising 19 converged algorithms. We further note that all algorithms in sets **A** and **B** yield a fractional error Δ*E*_F_ ≤ 0.005 (0.5%) for >99.8% of the 1500 total eigenstate predictions they are tested on. The distribution of these errors can be seen in Figure 6, where the upper panel includes the fractional errors of target-function trained algorithms in set **A**, and the lower panel shows those of the variationally trained algorithms in set **B**. Again, we see that many of the fractional errors are significantly below the 0.005 fractional-error threshold. Furthermore, we again note that the fractional errors of the variationally trained algorithms in set **B** are not only significantly lower but also demonstrate a narrower distribution.

**Figure 7:**
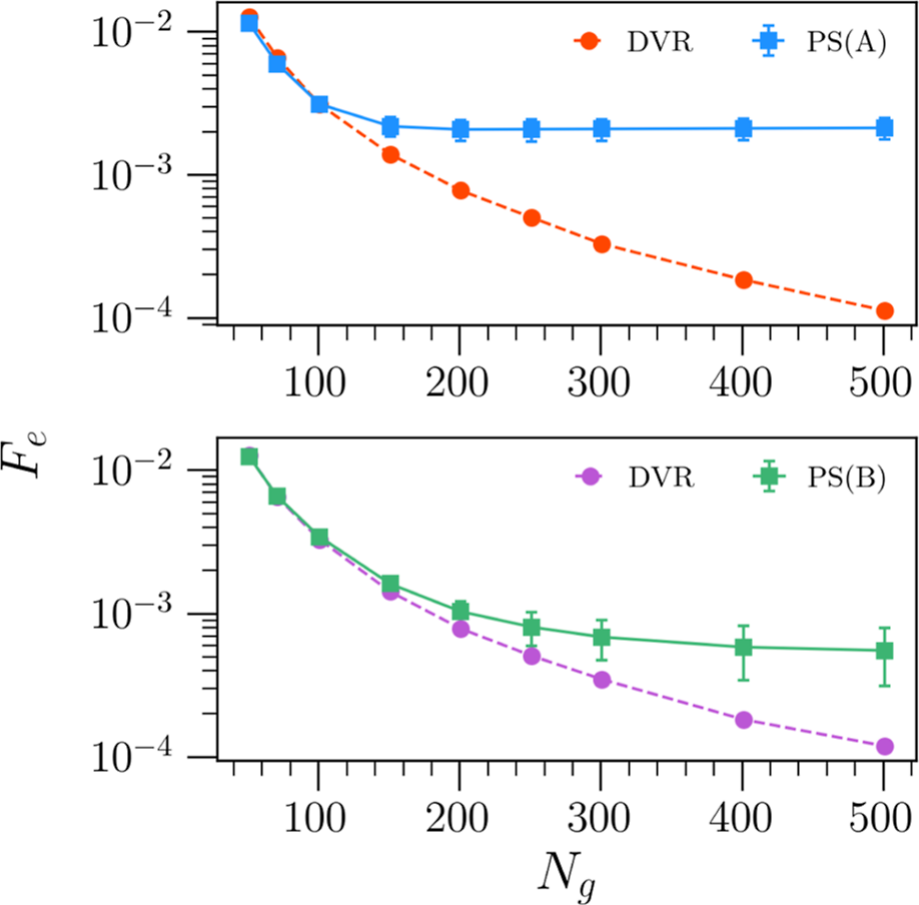
Convergence of the RMSE target function as a function of grid size for the set of algorithms trained using the target function (A) and the set that results from variational optimization (B). Note, shown are the averages of the RMSE over all algorithms in each set. Moreover, the larger lower error bars are only an artifact of the nonlinear logarithmic scale used. At each grid point, the algorithms in both sets predict the first three eigenstate energies of a test set comprising 500 random polynomial potentials.

Turning to Figure 7, we show the convergence of the target function with respect to the grid-size, averaged over all algorithms in sets **A** (upper panel) and **B** (lower panel). For all data points, the reference energies  (eq 4) are provided by the oracle DVR algorithm with *N*_g_ = 1001 grid points. Generally, both sets of algorithms result in accurate energy predictions across all grid sizes, with PS algorithms marginally beating the convergence of the DVR algorithm below *N*_g_ = 101. However, the convergence of the PS algorithms then levels off at larger grid-sizes. Crucially, although the convergence plateaus, the magnitude of the remaining errors is very small at larger grid-sizes (noting the logarithmic scale used in Figure 7). Comparing the two panels in Figure 7, the set of algorithms synthesized via pseudovariational optimization (**B**) appear to more closely track the convergence of the oracle DVR algorithm at larger grid-sizes. This suggests that the pseudovariational optimization is capable of identifying algorithms that are marginally better-performing than those algorithms generated by target-function optimization, as also suggested by Figure 6. Furthermore, despite training using just the single lowest-energy eigenstate for each training PES, the pseudovariational algorithms also predict accurate energies for higher eigenstates, at least in the case of a single DOF considered here.

#### 3.3.2 Extension to Higher Dimensions

Having identified a set of PS-generated algorithms that reliably converge in one dimension and exhibit transferability to a wide set of PESs, we proceeded to test the convergence of our algorithms on a further set of 50 two-dimensional PESs. Within this test-set, we include 10 PESs comprising both 2D isotropic and anisotropic harmonic oscillators as well as 40 potentials formed from a combination of Morse and asymmetric/symmetric double-well potentials coupled to harmonic oscillators.

**Figure 8:**
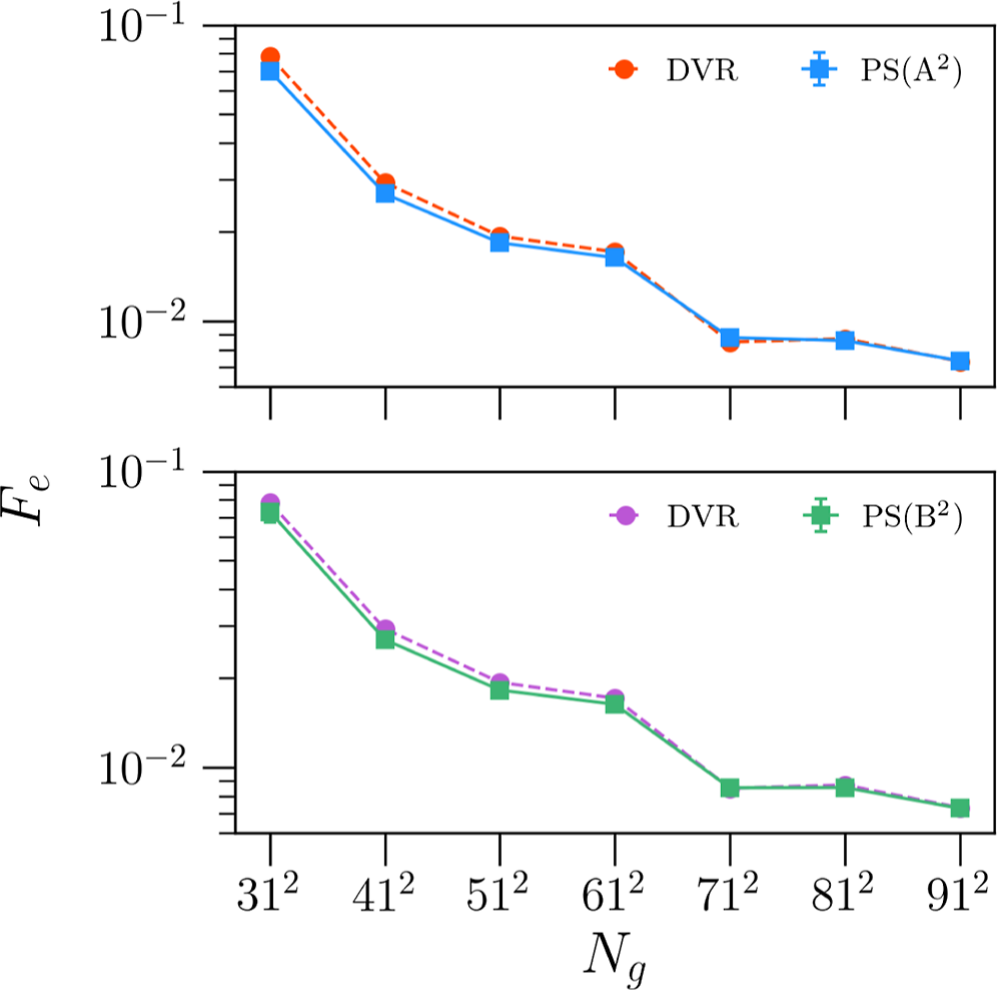
Convergence of the RMSE target function as a function of grid size in 2D for the set of algorithms trained using the target function (**A**^2^) and the set that results from variational optimization (**B**^2^). Note, we again plot the averages over the whole set of PS algorithms; however, the error bars are too small to be seen. At each grid-size, the algorithms in both sets predict the energies of the first three eigenstates for a test-set comprising 50 various 2D potentials.

The convergence of the target function as a function of grid-size for the set of 2D problems is shown in Figure 8. Here, the values are calculated with respect to the first three energy eigenvalues obtained from CM-DVR at a large grid-size of *N*_g_ = 101 × 101. Note, we only consider algorithms with fractional errors Δ*E*_F_ ≤ 0.005 over the set of 2D test problems. As such, set **A**^2^ (upper panel) corresponds to three 2D converged algorithms trained using the target function, whereas set **B**^2^ (bottom panel) contains six algorithms obtained from variational optimization. The values of the target function shown in the figure are averaged over each set of 2D converged algorithms. In both cases, the convergence of PS-generated algorithms closely tracks that of CM-DVR. Interestingly, at lower grid sizes in the range *N*_g_ ∈ [31^2^, 61^2^], the convergence of the PS-generated algorithms marginally outperforms that of CM-DVR; however, this small improvement decreases at larger grid sizes.

#### 3.3.3 Computational Performance Gains

Next, we proceeded to evaluate the gains in computational performance for our converged PS-generated algorithms relative to a traditional DVR algorithm. Naturally the computational resources required depend on both the number of DOFs (*d*) and the chosen size of the direct-product grid (*N*_g_). In testing performance, we generate a series of harmonic oscillator PESs up to *d* = 4, with each PES represented on a direct-product grid of *N*_g_ = *n*^*d*^ total grid points, where *n* ∈ [21, 25, 31]. For simplicity, we use a constant value of *n* along each of the *d* DOFs.

**Figure 9:**
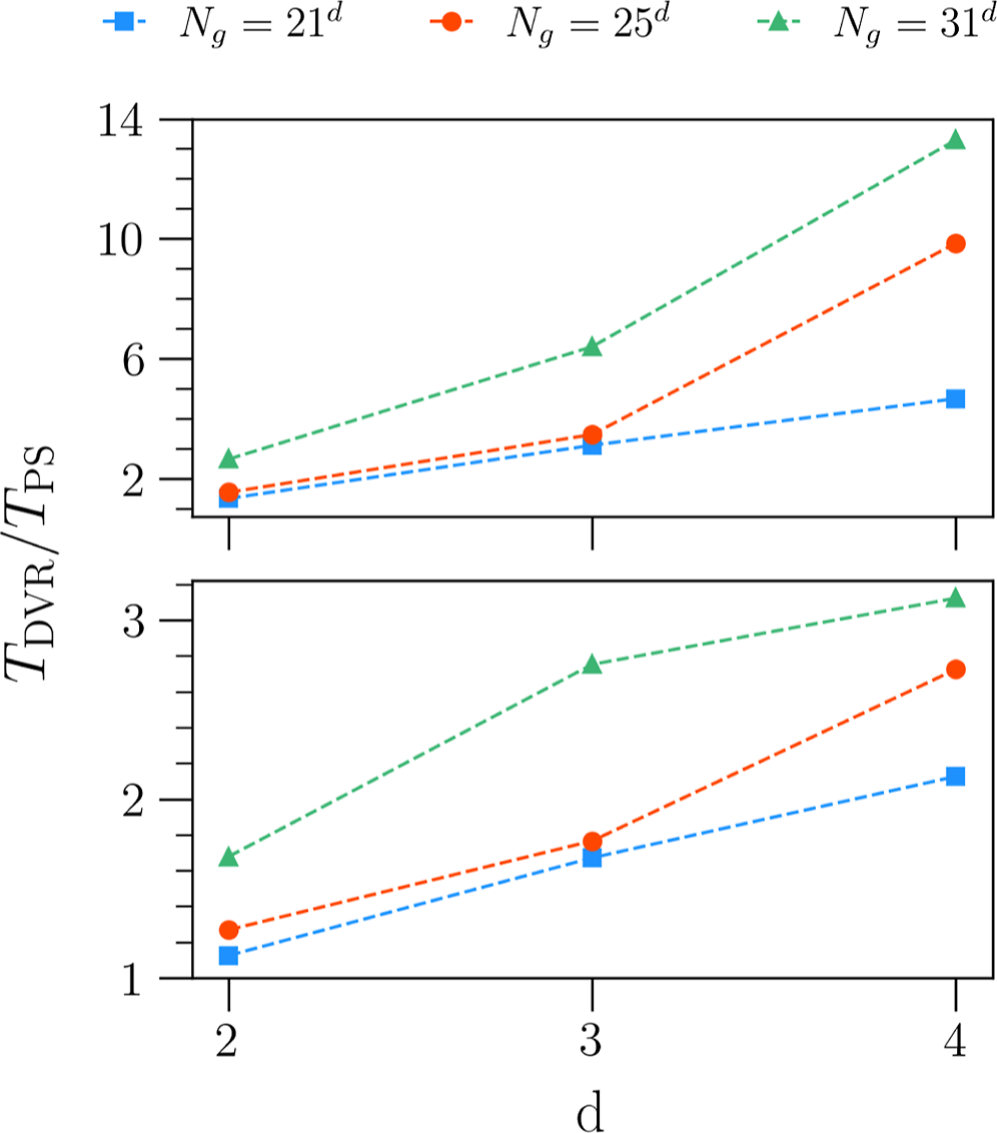
Speed-up (maximum of ten independent runs) in calculating the first three eigenstates of a *d*-dimensional harmonic oscillator in the full matrix representation of the Hamiltonian (upper panel) and using the matrix-vector product (lower panel). Here, each potential is represented on a product grid with *N*_g_ = *n*^*d*^ grid points, where *n* ∈ [21, 25, 31].

In testing the speed-up of PS-generated algorithms relative to CM-DVR, we compare the time taken to calculate the first three eigenstates of each *d*-dimensional harmonic oscillator. To get a measure of speed-up that is unbiased to parallelization gains, each computation was run in serial on an Intel i7-1300H CPU. In addition, we take the maximum speed-up recorded over 10 independent program runs. In the upper panel of Figure 9, we see the speed-up obtained when allocating the full Hamiltonian matrix in memory, using sparse matrix data structures and eigensolvers. Here, we observe that for smaller grid sizes of 21^*d*^ and 25^*d*^, the speed-up is initially just below a factor of 2. As the number of DOFs increases, we see that the increase in speedup is more significant for the larger grid-sizes, reaching a maximum of around 13 in the largest case. Generally, as the number of DOFs increases, one cannot store the full Hamiltonian matrix directly in memory, even in a sparse-storage format. For this reason, we also show the speed-up obtained by our PS-generated algorithms when using an iterative matrix-vector eigensolver in the lower panel of Figure 9. In this case, we observe a smaller but still significant speed-up, which comes solely from the sparse multiplication of each tridiagonal 1D KE matrix with the set of *n*^*d*–1^ subvectors, as previously outlined in Section 2.3.1. Here, the speed-up for a system with 2 DOFs is marginal at smaller grid sizes, becoming more beneficial as the number of DOFs increases. However, we still observe a significant speed-up (greater than a factor of 3) for the largest grid-size *N*_g_ = 31^4^.

**Figure 10:**
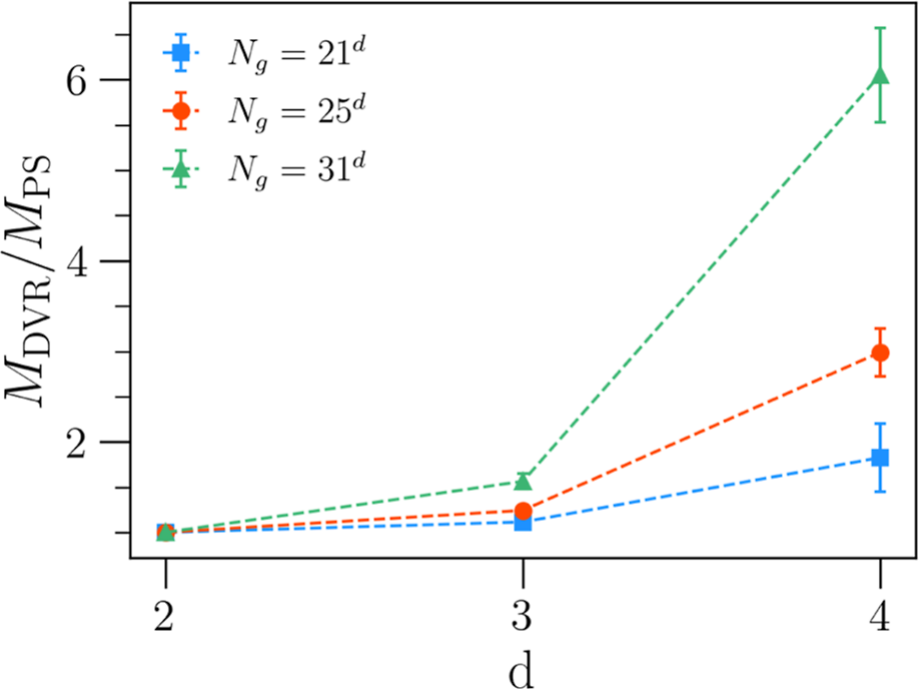
Ratio of the average maximum memory consumption (over 10 runs) for the oracle DVR algorithm (*M*_DVR_) vs an example PS algorithm (*M*_PS_) to calculate the first three eigenstates of a *d* dimensional harmonic oscillator in the full matrix representation of the Hamiltonian.

Despite still achieving a significant speed-up when using iterative matrix-vector eigensolvers, the savings in memory consumption are essentially negligible as the full Hamiltonian is never stored in memory. We show the savings in memory achieved by our PS algorithms when using the full matrix representation in Figure 10. In the case of the largest grid (*N*_g_ = 31^4^), we achieve a memory saving by a factor of 6. However, we note that in applications to tetra-atomic molecules or larger, one must use an iterative matrix-vector method.

### 3.4 Optimized Algorithms

Prior to exploring molecular applications, we shall proceed with a brief discussion of the variety of optimized algorithms. The KE matrix elements of each algorithm are defined in full in the Supporting Information. Crucially, the 1D KE matrix for a given DOF is always tridiagonal. In contrast, the full potential matrix is always diagonal and is given by the values of the PES at the grid points.

Importantly, for many of the discovered algorithms, parallels can be drawn with the matrix elements, as defined by CM-DVR (also given in Supporting Information). For example, all algorithms include operations involving the grid spacing along a DOF (d*x*), and many of these include division by a power of d*x* on the off-diagonal. Moreover, those that include division by d*x* on the diagonal tend to also include division by larger factors of d*x* on the off-diagonal. For example, algorithm A1, whose KE matrix elements are defined as
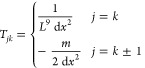
10includes division by d*x*^2^ on the diagonal and division by a factor of 2 × d*x*^2^ on the off-diagonal. This is to be expected as the magnitude of KE matrix elements decreases as one strays from the diagonal. In addition to this, many algorithms also include various factors or powers of the grid length/range (*L*), and the mass of the DOF (*m*), although in mass-weighted coordinates, the latter reduces to unity.

Perhaps somewhat more interestingly, we also discover algorithms that include various unexpected function operations, as with our previous work,^17,18^ for example, algorithm A2, whose KE matrix elements are defined as
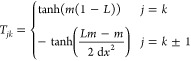
11which includes a hyperbolic tangent function. Similarly, a number of other algorithms (defined in the Supporting Information) contain a variety of trigonometric, hyperbolic, and logarithmic functions. However, these algorithms also include some combination of the more expected operations. Again, this further supports the conclusion that we are able to discover algorithms that correspond to functions of the Hamiltonian, which yield the same set of eigenvectors.

### 3.5 Applications to Molecular Systems

Having generated, tested, and validated the performance of 400 total different PS-generated algorithms for model PESs, we now turn to assessing the performance of these algorithms in predicting vibrational spectra for real molecular systems. These results represent the first-ever application of computer-generated algorithms to address a “real-world” prediction for molecular systems and highlight the potential for future PS applications in the quantum chemical sciences.

#### 3.5.1 Triatomic Examples

Here, we used the converged PS-generated algorithms to calculate the vibrational transition energies of H_2_O, NO_2_, and SO_2_. In each case, calculations were performed as outlined in Section 2.3.1. Taking the combined set of nine 2D converged algorithms from sets **A**^2^ and **B**^2^, we find a total of seven PS algorithms that yield reliable molecular predictions, corresponding to transition energies that agree with both CM-DVR and sine-DVR calculations largely within 1 cm^–1^.

The first nine vibrational transition energies from CM-DVR and PS-generated algorithm A1 (eq 10) are given in Table 1; the full table of the first 20 experimental and calculated vibrational transitions can be found in the Supporting Information. From Table 1, it is clear that algorithm A1 predicts transition energies in close agreement to CM-DVR across all three molecular examples, largely within subwavenumber (<1 cm^–1^) accuracy.

**Table 1:** First Nine Vibrational Transitions (Ordered in Increasing Energy) for H_2_O, NO_2_, and SO_2_, Calculated Using CM-DVR and PS Algorithm A1^a^

transition	H_2_O		NO_2_		SO_2_	
	CM	PS (A1)	CM	PS (A1)	CM	PS (A1)
1	1581.60	1581.63	758.28	758.35	517.45	517.48
2	3125.04	3125.17	1365.10	1365.30	1034.62	1034.60
3	3655.76	3655.81	1515.88	1516.18	1164.62	1165.17
4	3741.69	3741.80	1643.80	1644.30	1357.94	1359.53
5	4626.47	4626.78	2116.98	2117.27	1552.04	1552.72
6	5221.22	5221.29	2272.64	2273.46	1679.27	1679.87
7	5279.35	5279.51	2391.13	2391.72	1870.89	1872.55
8	6079.62	6080.29	2719.95	2718.17	2072.29	2068.94
9	6748.54	6748.69	2868.04	2868.58	2193.73	2194.22

a All transitions are given in cm^–1^.

**Figure 11:**
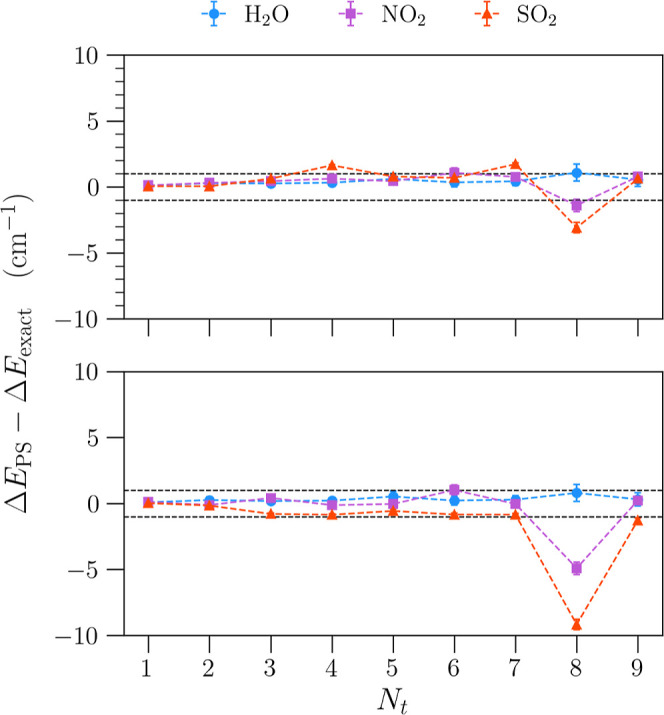
Average difference between vibrational transition energies calculated from seven PS algorithms and two existing DVR algorithms, CM-DVR (upper panel) and sine-DVR (lower panel). Here, we show the first nine transitions for the set of three triatomic molecules. The large majority of PS-predicted transitions are within 1 cm^–1^, as indicated by the dashed black lines.

To further demonstrate that all seven converged algorithms yield reliable predictions, we plot the average difference of the first nine transition energies from the predictions of CM-DVR in the top panel of Figure 11. Furthermore, we plot the difference relative to sine-DVR in the bottom panel of the figure. Here, we see that the predictions are largely within 1 cm^–1^ of both CM-DVR and sine-DVR, as indicated by the dashed black lines at ±1 cm^–1^. However, the eighth transition for NO_2_ and SO_2_ appears to deviate from the predictions of CM-DVR and sine-DVR (albeit still within a small error of 3 and 10 cm^–1^ for CM-DVR and sine-DVR, respectively). Here, we note that while our PS algorithms are slightly further from the predictions of CM-DVR and sine-DVR, they are in fact marginally closer to the experimental values for that particular transition (see Tables S6 and S9 in the Supporting Information). However, it is difficult to rule out that this is not an effect of grid-size since different algorithms converge at different rates. Importantly, in both panels, we see that the standard deviations among the seven PS algorithms are small, with all PS algorithms in close agreement. The full list of Hamiltonian matrix elements for each of these algorithms are given in the Supporting Information.

#### 3.5.2 Improving Sampling

The calculations described above employed full direct-product grids, with the relevant values of the PES for each triatomic molecule evaluated directly at each grid-point. As noted above, and in our previous studies using MCTDH,^5,51,52,54^ we have shown how ML strategies such as GPR can be readily adapted to generate accurate *semiglobal* PESs but using fewer PES evaluations than usually would be required. In addition, we have also highlighted the benefits of using nondirect-product grids in sampling configurations for PES generation using GPR;^5,51,52^ here, we test the combination of GPR, Sobol sequence sampling of configurations for PES construction, and PS-generated algorithms to validate this approach for theoretical vibrational spectroscopy.

**Figure 12:**
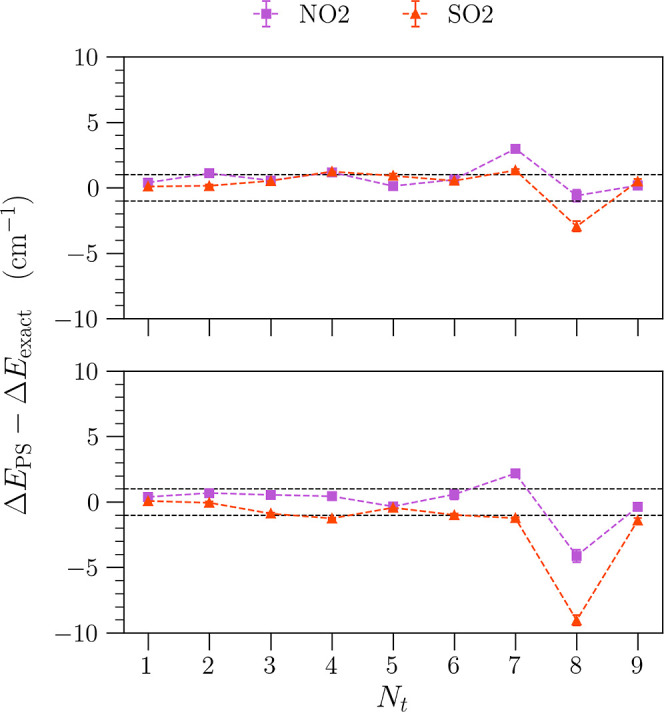
Average differences between PS-predicted transition energies calculated using a GPR-PES (fit using 128 Sobol points), and transition energies predicted by CM-DVR (top) and sine-DVR (bottom) using pure DFT potentials.

In Figure 12, we show the average difference between PS-calculated transition energies using the GPR-PES (sampled with 128 points generated by Sobol sequence sampling) compared to the transition energies calculated by CM-DVR (upper panel) or sine-DVR (lower panel) using the full direct-product DFT PES. In both panels, we find that the majority of transitions are still within 1 cm^–1^ of the standard DVR results obtained using full direct-product grids. Compared to Figure 11, we also note some deviations from the PS-predicted transitions using the full direct-product DFT PES; however, these are generally small in magnitude. Importantly, we note that the number of ab initio calculations required in the GPR-based calculations was just 128, much smaller than that required on the full direct-product grid (39,401). Furthermore, in the case of SO_2_, we find that we can reduce the number of Sobol sampling points to 64 and still retain predicted vibrational transitions with subwavenumber accuracy relative to CM-DVR. This can be seen in Figure S4 in the Supporting Information. As such, given that only a small fraction of ab initio calculations are required to accurately reproduce the transition lines using GPR-based PS-generated algorithms and the PS-created algorithms are also inherently efficient due to sparse structure, this is a particularly encouraging result.

## 4 Conclusions

In this article, we have introduced a new PS framework to facilitate the generation of DVR-like algorithms that solve the vibrational Schrödinger equation. In contrast to previous work,^17,18^ the approach taken here ensures separability of the kinetic energy and potential energy operator matrices by assuming that the potential energy matrix is diagonal in the assumed underlying wave function representation; however, we note that no explicit basis functional form is employed, with PS instead free to generate kinetic energy matrix representations that optimize predictive performance. Furthermore, because of the simple factorization of the KEO operator matrices, this approach is trivially extendable to higher dimensions, just like existing DVR algorithms. We have demonstrated that, by enforcing a tridiagonal kinetic matrix structure, additional savings in computational resources are achieved. Although savings in memory are negligible when moving to iterative matrix-vector eignsolvers in higher dimensions, we are still able to achieve a speed-up of around a factor of 3 (relative to standard DVR calculations), as demonstrated for four-dimensional problems. In higher dimensions, this speed-up is expected to increase further. The speed-up results solely from the fact that PS-generated algorithms are defined by tridiagonal 1D kinetic energy matrices, each of which are multiplied by a series of *n*^*d*–1^ subvectors through a series of tensor transformations.

We also introduced a pseudovariational approach to algorithm generation in PS. In contrast to our previous reports, this strategy does not require numerically exact reference data from an oracle algorithm such as CM-DVR or sine-DVR and instead minimizes an energy vector with elements corresponding to the predicted ground-state energy of a given PES problem-set. Both the pseudovariational and standard target-function-based approaches were shown to yield a number of algorithms with good levels of performance.

In this article, all PS-created algorithms that exhibited an initial training performance below a user-defined threshold were subsequently subjected to a three-step testing and validation procedure. First, although algorithms were trained on a range of 1D grid sizes, we explicitly checked the energy convergence of all algorithms with respect to the grid-size, removing algorithms that yielded either diverging energies in the large grid limit or predicted unequal probability amplitude in symmetric double-well potentials. Second, the remaining algorithms were tested on a range of 500 randomly generated 1D bound-polynomial PESs. Finally, those algorithms that performed well in both preceding steps were tested in 2D. This resulted in a total of nine algorithms identified as being sufficiently accurate for further applications (as shown in Supporting Information).

From these nine algorithms, seven were found to accurately predict the first nine vibrational transitions of H_2_O, NO_2_, and SO_2_ largely within 1 cm^–1^ of the two reference DVR algorithms. Crucially, this demonstrates the first successful application of PS-generated algorithms to molecular systems. In addition, we have shown that the predicted transitions are largely unaffected when using a GPR-PES trained on Sobol-sampled points in the configuration space. This significantly reduces the number of ab initio calculations from 39,401 to 128. We envision that this approach may be useful in sampling the configuration space for fitting accurate spectroscopic potentials, which typically involves fitting a functional form to a combination of high-level ab initio calculations and experimental data.

While we assume a diagonal potential energy operator and a system of normal coordinates to facilitate the extension of algorithms trained in 1D to higher dimensions, this is not necessarily a requirement. We note that systems with fast vibrational motion may be better described by alternative coordinate systems, in which the KEO is not separable. Here, one could envision synthesizing algorithms for a specific number of DOFs, although this would greatly increase the cost of training in higher dimensions. Moreover, although the direct product DVR representation employed in this work simplifies the computation of the potential matrix, beyond six DOFs one quickly runs into issues. Alternatively, one could envision reformulating our PS scheme to operate on the FBR basis where the potential matrix is no longer diagonal. In such a setup, one could utilize an *n*-mode representation of the potential and generate algorithms that approximate the potential matrix. Typically, such representations require fewer grid points for convergence and can be more readily applied to systems larger than six DOFs.^31^

To conclude, we note that the successful application of PS-generated algorithms to molecular systems reported in this article holds great promise for future applications of PS in various other fields, including electronic structure theory, semiclassical trajectory-based dynamics, and Gaussian wave packet approaches to quantum dynamics. These are all now areas of active interest in this research direction. Perhaps in these more complicated applications of PS, the discovered algorithms may uncover additional physical insight that could be exploited and adapted in human-developed algorithms.

## Data Availability

The data used to generate Figures 2–12 is available on the Warwick Research Archive Portal at wrap.warwick.ac.uk/188012.

## Supporting Information Available

Further details of DVR calculation methods employed, calculation of molecular vibrational spectra, PS functional library details, and mathematical representations of the final high-performing algorithms (PDF)

## References

[ref1] Dral P. O. Quantum Chemistry in the Age of Machine Learning. J. Phys. Chem. Lett. 2020, 11, 2336–2347. 10.1021/acs.jpclett.9b03664. 32125858

[ref2] Dral P. O.; Owens A.; Dral A.; Csányi G. Hierarchical machine learning of potential energy surfaces. J. Chem. Phys. 2020, 152, 204110 10.1063/5.0006498. 32486656

[ref3] Deringer V. L.; Caro M. A.; Csányi G. Machine Learning Interatomic Potentials as Emerging Tools for Materials Science. Adv. Mater. 2019, 31, 1902765 10.1002/adma.201902765. 31486179

[ref4] Manzhos S. Machine learning for the solution of the Schrödinger equation. Mach. Learn.: Sci. Technol. 2020, 1, 013002 10.1088/2632-2153/ab7d30.

[ref5] Richings G. W.; Habershon S. Predicting Molecular Photochemistry Using Machine-Learning-Enhanced Quantum Dynamics Simulations. Acc. Chem. Res. 2022, 55, 209–220. 10.1021/acs.accounts.1c00665. 34982533

[ref6] Westermayr J.; Marquetand P. Machine Learning for Electronically Excited States of Molecules. Chem. Rev. 2021, 121, 9873–9926. 10.1021/acs.chemrev.0c00749. 33211478 PMC8391943

[ref7] Li J.; Lopez S. A. A Look Inside the Black Box of Machine Learning Photodynamics Simulations. Acc. Chem. Res. 2022, 55, 1972–1984. 10.1021/acs.accounts.2c00288. 35796602

[ref8] Deringer V. L.; Bartók A. P.; Bernstein N.; Wilkins D. M.; Ceriotti M.; Csányi G. Gaussian Process Regression for Materials and Molecules. Chem. Rev. 2021, 121, 10073–10141. 10.1021/acs.chemrev.1c00022. 34398616 PMC8391963

[ref9] Kolb B.; Marshall P.; Zhao B.; Jiang B.; Guo H. Representing Global Reactive Potential Energy Surfaces Using Gaussian Processes. J. Phys. Chem. A 2017, 121, 2552–2557. 10.1021/acs.jpca.7b01182. 28287725

[ref10] Smith J. S.; Roitberg A. E.; Isayev O. Transforming Computational Drug Discovery with Machine Learning and AI. ACS Med. Chem. Lett. 2018, 9, 1065–1069. 10.1021/acsmedchemlett.8b00437. 30429945 PMC6231187

[ref11] Wengert S.; Csányi G.; Reuter K.; Margraf J. T. Data-efficient machine learning for molecular crystal structure prediction. Chem. Sci. 2021, 12, 4536–4546. 10.1039/D0SC05765G. 34163719 PMC8179468

[ref12] Schmidt M.; Lipson H. Distilling Free-Form Natural Laws from Experimental Data. Science 2009, 324, 81–85. 10.1126/science.1165893. 19342586

[ref13] Brunton S. L.; Proctor J. L.; Kutz J. N. Proc. Natl. Acad. Sci. U.S.A. 2016, 113, 3932–3937. 10.1073/pnas.1517384113. 27035946 PMC4839439

[ref14] Udrescu S.-M.; Tegmark M. AI Feynman: A physics-inspired method for symbolic regression. Sci. Adv. 2020, 6, eaay2631 10.1126/sciadv.aay2631. 32426452 PMC7159912

[ref15] Makke N.; Chawla S. Interpretable scientific discovery with symbolic regression: a review. Artif. Intell. Rev. 2024, 57, 2 10.1007/s10462-023-10622-0.

[ref16] Rudin C. Stop explaining black box machine learning models for high stakes decisions and use interpretable models instead. Nat. Mach. Intell. 2019, 1, 206–215. 10.1038/s42256-019-0048-x. 35603010 PMC9122117

[ref17] Habershon S. Solving the Schrödinger equation using program synthesis. J. Chem. Phys. 2021, 155, 154102 10.1063/5.0062497. 34686051

[ref18] Habershon S. Program Synthesis of Sparse Algorithms for Wave Function and Energy Prediction in Grid-Based Quantum Simulations. J. Chem. Theory Comput. 2022, 18, 2462–2478. 10.1021/acs.jctc.2c00035. 35293216 PMC9009083

[ref19] Manzhos S.; Yamashita K.; Carrington T. Using a neural network based method to solve the vibrational Schrödinger equation for H2O. Chem. Phys. Lett. 2009, 474, 217–221. 10.1016/j.cplett.2009.04.031.

[ref20] Manzhos S.; Carrington T. An improved neural network method for solving the Schrödinger equation. Can. J. Chem. 2009, 87, 864–871. 10.1139/v09-025.

[ref21] Colbert D. T.; Miller W. H. A novel discrete variable representation for quantum mechanical reactive scattering via the *S* -matrix Kohn method. J. Chem. Phys. 1992, 96, 1982–1991. 10.1063/1.462100.

[ref22] Tannor D. J. Introduction to Quantum Mechanics: A Time-Dependent Perspective; University Science Books, 2007.

[ref23] Harris D. O.; Engerholm G. G.; Gwinn W. D. Calculation of Matrix Elements for One-Dimensional Quantum-Mechanical Problems and the Application to Anharmonic Oscillators. J. Chem. Phys. 1965, 43, 1515–1517. 10.1063/1.1696963.

[ref24] Light J. C.; Carrington T. Adv. Chem. Phys. 2000, 114, 263–310. 10.1002/9780470141731.ch4.

[ref25] Wei H. Ghost levels and near-variational forms of the discrete variable representation: Application to H2O. J. Chem. Phys. 1997, 106, 6885–6900. 10.1063/1.473714.

[ref26] Bowman J. M.; Gazdy B. A truncation/recoupling method for basis set calculations of eigenvalues and eigenvectors. J. Chem. Phys. 1991, 94, 454–460. 10.1063/1.460361.

[ref27] Cooper J.; Carrington T. Computing vibrational energy levels by using mappings to fully exploit the structure of a pruned product basis. J. Chem. Phys. 2009, 130, 214110 10.1063/1.3140272. 19508059

[ref28] Bramley M. J.; Handy N. C. Efficient calculation of rovibrational eigenstates of sequentially bonded four-atom molecules. J. Chem. Phys. 1993, 98, 1378–1397. 10.1063/1.464305.

[ref29] Bačić Z.; Light J. C. Theoretical Methods for Rovibrational States of Floppy Molecules. Annu. Rev. Phys. Chem. 1989, 40, 469–498. 10.1146/annurev.pc.40.100189.002345.

[ref30] Bowman J. M.; Carrington T.; Meyer H.-D. Variational quantum approaches for computing vibrational energies of polyatomic molecules. Mol. Phys. 2008, 106, 2145–2182. 10.1080/00268970802258609.

[ref31] Carrington T. Iterative Methods for Computing Vibrational Spectra. Mathematics 2018, 6, 13 10.3390/math6010013.

[ref32] Partridge H.; Schwenke D. W. J. Chem. Phys. 1997, 106, 4618–4639. 10.1063/1.473987.

[ref33] Marquardt R.; Quack M. Global analytical potential hypersurfaces for large amplitude nuclear motion and reactions in methane. I. Formulation of the potentials and adjustment of parameters to ab initio data and experimental constraints. J. Chem. Phys. 1998, 109, 10628–10643. 10.1063/1.476513.

[ref34] Marquardt R.; Quack M. Global Analytical Potential Hypersurface for Large Amplitude Nuclear Motion and Reactions in Methane II. Characteristic Properties of the Potential and Comparison to Other Potentials and Experimental Information. J. Phys. Chem. A 2004, 108, 3166–3181. 10.1021/jp037305v.

[ref35] Marquardt R.; Quack M. Handbook of High-Resolution Spectroscopy; John Wiley & Sons, Ltd, 2011.

[ref36] Marquardt R.; Sagui K.; Zheng J.; Thiel W.; Luckhaus D.; Yurchenko S.; Mariotti F.; Quack M. Global Analytical Potential Energy Surface for the Electronic Ground State of NH3 from High Level ab Initio Calculations. J. Phys. Chem. A 2013, 117, 7502–7522. 10.1021/jp4016728. 23688044

[ref37] Kuhn B.; Rizzo T. R.; Luckhaus D.; Quack M.; Suhm M. A. A new six-dimensional analytical potential up to chemically significant energies for the electronic ground state of hydrogen peroxide. J. Chem. Phys. 1999, 111, 2565–2587. 10.1063/1.479534.

[ref38] Huang X.; Schwenke D. W.; Lee T. J. An accurate global potential energy surface, dipole moment surface, and rovibrational frequencies for NH3. J. Chem. Phys. 2008, 129, 214304 10.1063/1.3025885. 19063558

[ref39] Zhang X.; Zou S.; Harding L. B.; Bowman J. M. A Global ab Initio Potential Energy Surface for Formaldehyde. J. Phys. Chem. A 2004, 108, 8980–8986. 10.1021/jp048339l.

[ref40] Jin Z.; Braams B. J.; Bowman J. M. An ab Initio Based Global Potential Energy Surface Describing CH5+ → CH3+ + H2. J. Phys. Chem. A 2006, 110, 1569–1574. 10.1021/jp053848o. 16435818

[ref41] Shepler B. C.; Braams B. J.; Bowman J. M. “Roaming” Dynamics in CH3CHO Photodissociation Revealed on a Global Potential Energy Surface. J. Phys. Chem. A 2008, 112, 9344–9351. 10.1021/jp802331t. 18597443

[ref42] Braams B. J.; Bowman J. M. Permutationally invariant potential energy surfaces in high dimensionality. Int. Rev. Phys. Chem. 2009, 28, 577–606. 10.1080/01442350903234923. 29401038

[ref43] Avila G.; Carrington T. Using a pruned basis, a non-product quadrature grid, and the exact Watson normal-coordinate kinetic energy operator to solve the vibrational Schrödinger equation for C2H4. J. Chem. Phys. 2011, 135, 064101 10.1063/1.3617249. 21842920

[ref44] Avila G.; Carrington T. Solving the Schroedinger equation using Smolyak interpolants. J. Chem. Phys. 2013, 139, 134114 10.1063/1.4821348. 24116559

[ref45] Avila G.; Carrington T. Using multi-dimensional Smolyak interpolation to make a sum-of-products potential. J. Chem. Phys. 2015, 143, 044106 10.1063/1.4926651. 26233106

[ref46] Avila G.; Carrington T. Reducing the cost of using collocation to compute vibrational energy levels: Results for CH2NH. J. Chem. Phys. 2017, 147, 064103 10.1063/1.4994920. 28810786

[ref47] Avila G.; Carrington T. Computing vibrational energy levels of CH4 with a Smolyak collocation method. J. Chem. Phys. 2017, 147, 144102 10.1063/1.4999153. 29031264

[ref48] Zak E. J.; Carrington T. Using collocation and a hierarchical basis to solve the vibrational Schrödinger equation. J. Chem. Phys. 2019, 150, 204108 10.1063/1.5096169. 31153182

[ref49] Wodraszka R.; Carrington T. A pruned collocation-based multiconfiguration time-dependent Hartree approach using a Smolyak grid for solving the Schrödinger equation with a general potential energy surface. J. Chem. Phys. 2019, 150, 154108 10.1063/1.5093317. 31005102

[ref50] Sobol I. M. On the distribution of points in a cube and the approximate evaluation of integrals. USSR Comput. Math. Math. Phys. 1967, 7, 86–112. 10.1016/0041-5553(67)90144-9.

[ref51] Richings G. W.; Robertson C.; Habershon S. Improved on-the-Fly MCTDH Simulations with Many-Body-Potential Tensor Decomposition and Projection Diabatization. J. Chem. Theory Comput. 2019, 15, 857–870. 10.1021/acs.jctc.8b00819. 30521337

[ref52] Richings G. W.; Robertson C.; Habershon S. Can we use on-the-fly quantum simulations to connect molecular structure and sunscreen action?. Faraday Discuss. 2019, 216, 476–493. 10.1039/C8FD00228B. 31016309

[ref53] Lehoucq R. B.; Sorensen D. C.; Yang C. ARPACK Users’ Guide; Software, Environments, and Tools; Society for Industrial and Applied Mathematics, 1998; pp 43–66.

[ref54] Richings G. W.; Habershon S. Direct grid-based quantum dynamics on propagated diabatic potential energy surfaces. Chem. Phys. Lett. 2017, 683, 228–233. 10.1016/j.cplett.2017.01.063.

