# Supplementary material for: Exploring New Algorithms for Molecular Vibrational Spectroscopy Using Physics-Informed Program Synthesis
Source: J Chem Theory Comput. 2024 Dec 18;21(1):307–20. doi: 10.1021/acs.jctc.4c01312 (PMC11736792; doi:10.1021/acs.jctc.4c01312)
Supplement: Supplementary file 1 — ct4c01312_si_001.pdf [file ct4c01312_si_001.pdf]

Supporting Information: Exploring new algorithms for molecular vibrational spectroscopy using physics-informed program synthesis

Kyle Acheson and Scott Habershon*

Department of Chemistry, University of Warwick, Coventry CV4 7AL, United Kingdom

E-mail: S.Habershon@warwick.ac.uk

1 DVR

Discrete variable representation (DVR) methods involve numerically solving the Schrödinger equation by representing the wavefunction at a set of discrete points on a grid. To understand the DVR, we must first introduce the idea of the finite basis representation (FBR). Here the wavefunction is expanded in the basis of global continuous functions, which are typically classical orthogonal polynomials. Common polynomial's used include Chebyshev and Hermite polynomials, corresponding to a basis of particle in a box and harmonic oscillator functions respectively. The DVR approach is based on the Gaussian quadrature of these underlying polynomials, each of which have an associated weight function and set of quadrature points. Hence, if our basis of orthogonal polynomials are normalised by the root of the weight function as,

$$\phi_n\left(x\right) = \sqrt{w\left(x\right)}P_n\left(x\right), \tag{S1}$$

then the orthonormality relations are fulfilled. In this work we are mainly concerned with DVRs generated from diagonalization of the coordinate operator (or function of) in the basis of orthogonal polynomials, defined as,

$$X_{nm} = \int_a^b \phi_n^*\left(x\right) x \phi_m\left(x\right) dx. \tag{S2}$$

This is a tri-diagonal matrix due to three term recurrence relation of orthogonal polynomials. The matrix $\mathbf{X}$ is diagonalized by a unitary matrix of eigenvectors that defines a transformation between the FBR and DVR. In addition, the eigenvalues of the matrix $\mathbf{X}$ correspond to a set of quadrature points localised at x_a. The DVR functions that are somewhat localised around the values of x_a are thus defined as,

$$\theta_i\left(x_a\right) = \sum_j U_{ij}^*\phi_j\left(x\right), \tag{S3}$$

with U_{ij} the elements of the transformation matrix. These DVR functions can then be used to evaluate Hamiltonian matrix elements directly. For example, in the DVR basis the potential energy (PE) matrix is diagonal and the elements are given by the potential values at the quadrature points,

$$\begin{aligned} V_{ij}^{\mathrm{DVR}} &= \langle \theta_i | \, V(x) \, | \theta_j \rangle \\ &= V(x_a) \delta_{ij}, \end{aligned} \tag{S4}$$

which greatly simplifies the evaluation. In contrast, in the FBR the PE matrix is evaluated according to the underlying quadrature,

$$V_{ij}^{\mathrm{FBR}} = \sum_{a=1}^{n} w_a \phi_i^* \left(x_a \right) V \left(x_a \right) \phi_j \left(x_a \right), \tag{S5}$$

with w_a the weight at quadrature point x_a. In the following subsections, we provide an overview of the two DVR methods utilized in this work.

1.1 Sine-DVR

Sine-DVR is often referred to as a *'proper'* DVR, due to the fact that quadrature points and weights can be analytically derived. Here the underlying basis corresponds to a set of particle in a box functions,

$$\phi_j \left(x \right) = \sqrt{\frac{2}{L}} \sin \left(j \pi \left(x - x_0 \right) / L \right), \tag{S6}$$

with L the range spanned by the coordinate grid and x_0 its minimum value. In this case, we must diagonalize a function of the coordinate operator,

$$f(x) = \cos \left(\pi \left(x - x_0 \right) / L \right). \tag{S7}$$

Evaluating this in the basis of sine functions leads to the matrix,

$$F_{ij} = \frac{1}{2} \begin{bmatrix} 0 & 1 & 0 & \dots \\ 1 & 0 & 1 & \dots \\ 0 & 1 & 0 & \dots \\ \vdots & \vdots & \vdots & \ddots \end{bmatrix}, \tag{S8}$$

which can be diagonalized analytically with the matrix,

$$U_{ja} = \sqrt{\frac{2}{n+1}} \sin\left(\frac{ja\pi}{n+1}\right), \tag{S9}$$

yielding eigenvalues,

$$f_a = \cos\left(\frac{a\pi}{n+1}\right). \tag{S10}$$

Since we have diagonalized a function of the coordinate operator, we must transform these eigenvalues back to yield DVR grid points,

$$\begin{aligned} x_a &= f^{-1}(f_a) = x_0 + \frac{L}{\pi} \arccos(f_a) \\ &= x_0 + a\frac{L}{n+1} \\ &= x_0 + a\Delta x, \end{aligned} \tag{S11}$$

where $\Delta x = L/n + 1$ and $a = 1, 2, \dots n$. Here the grid is uniformly spaced with constant weights $w_a = \Delta x$. Interestingly, this corresponds to an underlying Gauss-Chebyshev quadrature of the second kind.

In this work, we evaluate the PE matrix in the DVR basis according to Eq. S4. However, the kinetic energy (KE) matrix is constructed in the FBR basis and then transformed to the

DVR. In the FBR the KE matrix elements are defined as,

$$\begin{aligned} T_{jk}^{\text{FBR}} &= -\frac{\hbar^2}{2m} \langle\phi_j| \frac{\partial^2}{\partial x^2} |\phi_k\rangle \\ &= \frac{\hbar^2}{2m} \left(\frac{j\pi}{L}\right)^2 \delta_{jk}. \end{aligned} \tag{S12}$$

This is transformed to the DVR basis as,

$$\mathbf{T}^{\text{DVR}} = \mathbf{U}^{\dagger}\mathbf{T}^{\text{FBR}}\mathbf{U}. \tag{S13}$$

The total Hamiltonian in the DVR basis can then be constructed and diagonalized to yield the wavefunctions and energy eigenvalues.

1.2 Colbert-Miller DVR

Colbert and Miller introduced a universal DVR in which the Hamiltonian matrix elements can be evaluated without explicitly defined basis functions. The KE operator was evaluated in the basis of particle in a box functions (Chebyshev polynomials). Assuming the number of functions goes to infinity as the range becomes infinite, the basis becomes an infinite set of sinc functions and the KE operator only depends on the distance between grid points. In this limit the KE matrix elements are,

$$T_{jk} = \begin{cases} \frac{\hbar^2\pi^2}{6m\ dx^2} & j = k \\ \frac{(-1)^{j-k}}{m\ dx^2 (j-k)^2} & j \neq k \end{cases} \tag{S14}$$

where dx corresponds to the spacing between equally spaced grid points x_i and x_j. In addition, the PE matrix is simply given by the values of the potential at each of the grid points. The advantage of CM-DVR lies in its simplicity, however in practical applications the KE is not given exactly due to the truncation of the basis.

1.3 Iterative-Matrix Methods

Iterative methods of the Lanczos variety allow one to solve the eigenvalue equation,

$$\mathbf{H}\phi = \mathbf{E}\phi, \tag{S15}$$

without having to store the whole Hamiltonian in memory. Such approaches require one to calculate the matrix-vector product $\mathbf{Hv}$ at each iteration. Given a d dimensional system represented on a direct product grid of n discrete points along each degree of freedom (DOF), the total size of the Hamiltonian is $n^d \times n^d$. In the naive approach to calculating $\mathbf{Hv}$, this requires n^{2d} total multiplications. However, exploiting the structure of the d-dimensional Hamiltonian, one can achieve significant speedup in calculating the matrix-vector product. Since, in the DVR basis the potential matrix is always diagonal the potential matrix-vector product can always be done in n^d multiplications. Thus, we shall exclude the potential from our discussion and focus on the d-dimensional kinetic matrix-vector multiplication.

In the direct product basis, a two dimensional KE matrix is defined as,

$$T_{ij,i'j'} = T_{ii'}\delta_{jj'} + T_{jj'}\delta_{ii'}. \tag{S16}$$

In the d-dimensional case this can be generalised to a Kronecker sum over a series of 1D KE matrices,

$$\mathbf{T} = \bigoplus_{k=1}^{d} \mathbf{T^k}, \tag{S17}$$

where each $\mathbf{T^k}$ is a 1D KE matrix of size $n \times n$, calculated for the kth DOF. To briefly demonstrate the calculation of the matrix-vector product, let us consider the two dimensional case assuming each $\mathbf{T^k}$ matrix is of size $n \times n$. In addition, let us define the vector $\mathbf{v}$ with elements $\mathbf{v} = (v_1, v_2, \ldots, v_{n^2})^{\text{T}}$. To calculate the product $\mathbf{Tv} = \mathbf{v}'$ first we multiply the kinetic matrix $\mathbf{T^1}$ by n sub-vectors of $\mathbf{v}$, each of length n. This set of n matrix-vector multiplications can be combined into a matrix multiplication by arranging the sub-vectors

in a matrix $\mathbf{V}$ of size $n \times n$ as,

$$\mathbf{T^1} \begin{bmatrix} v_1 & \dots & v_n \\ v_{n+1} & \dots & v_{2n} \\ v_{2n+1} & \dots & v_{3n} \\ \vdots & \ddots & \vdots \\ v_{n^2-n} & \dots & v_{n^2} \end{bmatrix} = \begin{bmatrix} y_1 & \dots & y_n \\ y_{n+1} & \dots & y_{2n} \\ y_{2n+1} & \dots & y_{3n} \\ \vdots & \ddots & \vdots \\ y_{n^2-n} & \dots & y_{n^2} \end{bmatrix}. \tag{S18}$$

The resulting matrix, containing the series of n sub-vector products of $\mathbf{v}$ is then flattened to give $\mathbf{y} = (y_1, y_2, \dots, y_{n^2})^{\mathrm{T}}$. In the second step, we perform a permutation of indices, which in the 2D case amounts to taking the transpose of the original matrix $\mathbf{V}$ in Eq. S18. We proceed to calculate the matrix-vector product $\mathbf{T^2}\mathbf{V}^{\mathrm{T}}$ as,

$$\mathbf{T^2} \begin{bmatrix} v_1 & v_{n+1} & v_{2n+1} & \dots & v_{n^2-n} \\ \vdots & \vdots & \vdots & \ddots & \vdots \\ v_n & v_{2n} & v_{3n} & \dots & v_{n^2} \end{bmatrix} = \begin{bmatrix} x_1 & x_{n+1} & x_{2n+1} & \dots & x_{n^2-n} \\ \vdots & \vdots & \vdots & \ddots & \vdots \\ x_n & x_{2n} & x_{3n} & \dots & x_{n^2} \end{bmatrix}, \tag{S19}$$

where the result of the products are organised in the matrix $\mathbf{X}$ on the right-hand side of the equation. Another permutation is performed on $\mathbf{X}$, which undoes the original index transformation applied to the matrix of sub-vectors $\mathbf{V}$. Again, this corresponds to the transposition $\mathbf{X}^{\mathrm{T}}$ in the 2D case. The matrix $\mathbf{X}^{\mathrm{T}}$ is then flattened along the rows to give the result of the vector product as $\mathbf{x} = (x_1, x_2, \dots, x_{n^2})^{\mathrm{T}}$. The total matrix-vector product $\mathbf{Tv} = \mathbf{v}'$ is simply given by addition of the matrix-vector products that result from the two individual steps as $\mathbf{v}' = \mathbf{x} + \mathbf{y}$.

In the more general case of a d-dimensional KE operator we treat the vector $\mathbf{v}$ of length n^d as a rank d tensor with a multi-index. For each of the k DOFs, we multiply each 1D KE matrix $\mathbf{T^k}$ by n^{d-1} sub-vectors of $\mathbf{v}$. At each iteration this requires a permutation of indices transforming the tensor into a matrix of shape $n \times n^{d-1}$, containing the n^{d-1} sub-vectors at the kth iteration down the columns. As in the 2D case, the resulting product undergoes

a second permutation of indices transforming the result contained in the matrix back into the original rank d tensor. This is then flattened, and the total matrix-vector product is calculated by simply summing each of these terms. As this process is repeated for a total of d times, the total running time is $O\left(dn^{d+1}\right)$.

2 Molecular Calculations

In this section we detail the representations of the potential for each of the three test molecules; H_2O, NO_2, and SO_2. In all cases the potential is represented on a direct product grid of mass-weighted normal coordinates. For NO_2 and SO_2 we obtain the equilibrium geometry by performing a geometry optimisation with very tight self-consistent field convergence. This was done in the ORCA software package, using the the TightSCF keyword for the optimisation. In addition, we give the full set of the first 20 vibrational transitions as calculated by CM-DVR, sine-DVR, and program synthesis algorithms, also comparing with experimental values.

2.1 H_2O

2.1.1 Potential Representation

In the case of H_2O we utilise the well known Partridge-Schwenke potential. Here the reference geometry corresponds to the equilibrium position, given in Cartesian coordinates in Table. S1.

Table S1 Cartesian coordinates of the equilibrium geometry of H_2O in Ångström.

Atom	x	y	z
O	0.00	0.00	0.00
H	0.95865	0.00	0.00
H	-0.237556	0.928750	0.00

The range of each normal coordinate along with the number of points (n_g) used to represent the potential are defined in Table. S2. Furthermore, one-dimensional cuts of the potential along each of the normal coordinates can be seen in Fig. S1.

Table S2 The range and number of grid points used along each normal coordinate for representing the H_2O potential.

DOF	q^{min}	q^{max}	n_g
q_1	-80	70	41
q_2	-50	25	31
q_3	-30	30	31

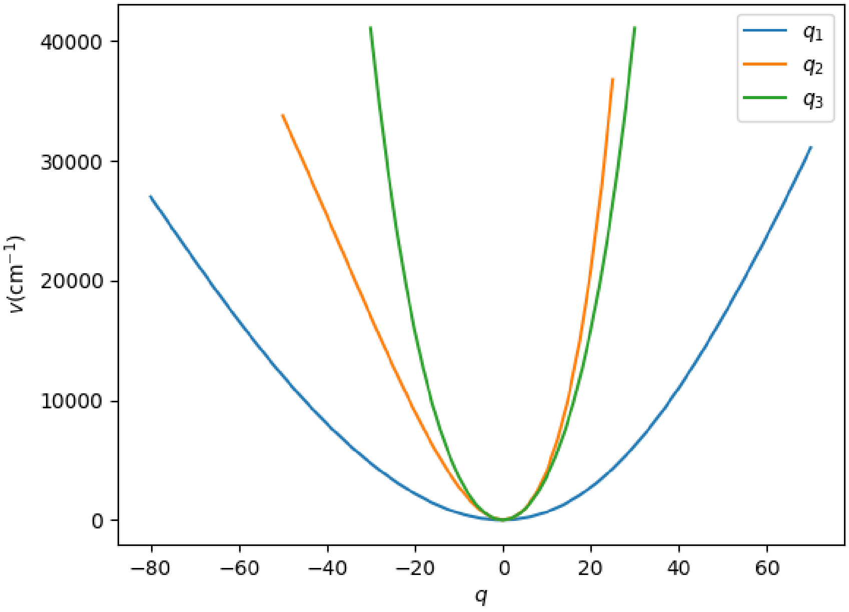

Figure S1 One-dimensional cuts of the H_2O potential over the range of normal coordinates used. For each DOF, one coordinate is varied while the other two are fixed at zero.

2.1.2 Vibrational Transitions

In Table. S3 we give the full table of the first 20 vibrational transitions energies and state assignments. Note that the transitions calculated by the PS algorithm A1, CM-DVR, and sine-DVR, are largely within $\approx 1\text{cm}^{-1}$ of each other. Although several select transitions show slightly larger variations. While calculated transitions energies vary by a larger amount in comparison to experiment, they broadly are within the experimental ballpark up to 8000cm^{-1}, above this limit the discrepancy becomes notably larger.

Table S3 Full table of the first 20 vibrational transition energies and assignments for H_2O. In the second column we show the experimental values, whereas columns 2-4 give the transitions calculated by CM-DVR, sine-DVR, and PS algorithm A1. Note all energies are given in cm^{-1}.

(v_1, v_2, v_3)	Exp.	CM-DVR	Sine-DVR	PS (A1))
(0,0,0)	0.00	0.00	0.00	0.00
(0,1,0)	1594.75	1581.60	1581.61	1581.63
(0,2,0)	3151.64	3125.04	3125.06	3125.17
(1,0,0)	3657.05	3655.76	3655.84	3655.81
(0,3,0)	3755.93	3741.69	3741.75	3741.80
(1,1,0)	4666.78	4626.45	4626.52	4626.78
(0,4,0)	5234.99	5221.23	5221.34	5221.29
(1,2,0)	5331.27	5279.35	5279.48	5279.51
(2,0,0)	6134.03	6079.62	6079.90	6080.29
(0,0,2)	6775.10	6748.54	6748.77	6748.69
(0,5,0)	6871.52	6781.03	6781.20	6781.31
(1,3,0)	7201.54	7197.02	7197.93	7196.67
(2,1,0)	7249.82	7235.74	7236.61	7236.52
(0,6,0)	7445.07	7420.44	7421.75	7419.40
(0,1,2)	8273.98	7474.82	7476.03	7475.92
(1,4,0)	8373.85	8234.37	8234.88	8234.72
(0,7,0)	8761.58	8243.10	8243.31	8243.60
(2,2,0)	8807.00	8738.06	8739.36	8737.76
(0,2,2)	9000.14	8757.39	8758.43	8758.34
(3,0,0)	9833.58	8796.74	8801.92	8798.21

2.2 NO_2

2.2.1 Potential Representation

For NO_2 we calculate the potential at the the B3LYP/def2-tzvp level of theory. The reference geometry corresponds to the equilibrium position, given in Cartesian coordinates in Table. S4.

Table S4 Cartesian coordinates of the equilibrium geometry of NO_2 in Ångström.

Atom	x	y	z
N	0.001011	0.002440	0.00
O	1.191918	-0.000971	0.00
O	-0.830153	0.855256	0.00

The range of each normal coordinate along with the number of points (n_g) used to

represent the potential are defined in Table. S5. Furthermore, one-dimensional cuts of the potential along each of the normal coordinates can be seen in Fig. S2.

Table S5 The range and number of grid points used along each normal coordinate for representing the NO_2 potential.

DOF	q^{min}	q^{max}	n_g
q_1	-80	80	41
q_2	-50	40	31
q_3	-40	40	31

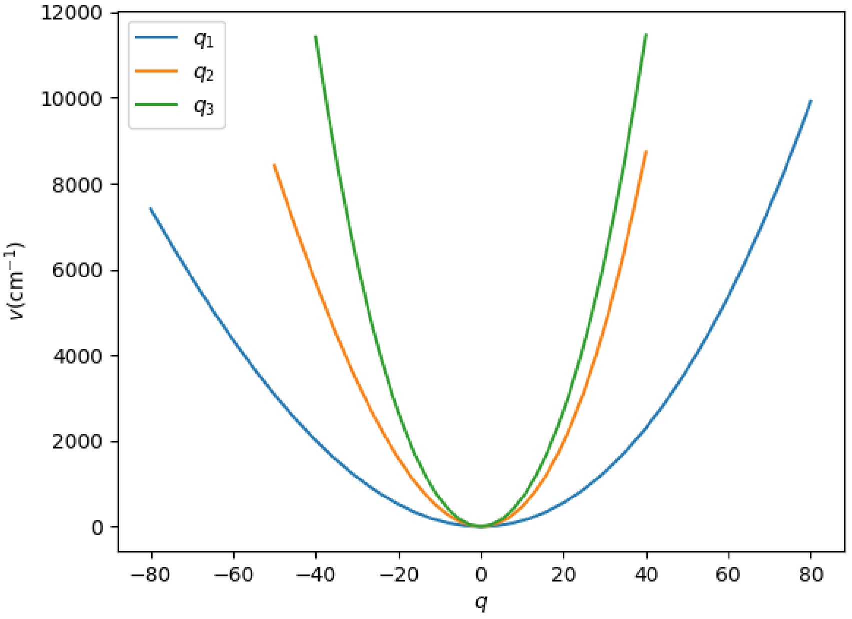

Figure S2 One-dimensional cuts of the NO_2 potential over the range of normal coordinates used. For each DOF, one coordinate is varied while the other two are fixed at zero.

2.2.2 Vibrational Transitions

Table. S6 tabulates the first 20 vibrational transitions energies and state assignments for NO_2. Again calculated transitions are given for PS algorithm A1, CM-DVR, and sine-DVR. These are also largely within $\approx 1\text{cm}^{-1}$ of each other, although larger variations are observed higher in the vibrational spectrum.

Table S6 Full table of the first 20 vibrational transition energies and assignments for NO_2. In the second column we show the experimental values, whereas columns 2-4 give the transitions calculated by CM-DVR, sine-DVR, and PS algorithm A1. Note all energies are given in cm^{-1}.

(v_1, v_2, v_3)	Exp.	CM-DVR	Sine-DVR	PS (A1))
(0,0,0)	0.00	0.00	0.00	0.00
(1,0,0)	749.659	758.28	758.29	758.35
(0,2,0)	1319.79	1365.10	1365.52	1365.30
(0,0,1)	1498.34	1515.88	1515.89	1516.18
(1,1,0)	1616.85	1643.80	1644.55	1644.30
(0,3,0)	2063.12	2116.98	2117.46	2117.27
(0,1,1)	2246.04	2272.64	2272.68	2273.46
(2,0,0)	2355.15	2391.13	2391.93	2391.72
(1,2,0)	2627.34	2719.95	2723.46	2718.17
(1,0,1)	2805.60	2868.04	2868.58	2868.58
(0,4,0)	2906.07	2979.25	2981.65	2980.71
(0,2,1)	2993.00	3028.48	3028.68	3030.12
(0,0,2)	3092.48	3137.78	3138.64	3138.62
(2,1,0)	3201.44	3260.25	3268.00	3255.08
(1,3,0)	3364.57	3465.55	3469.37	3463.68
(1,1,1)	3547.10	3618.09	3618.76	3619.24
(0,5,0)	3637.84	3720.06	3722.60	3721.68
(0,3,1)	3738.60	3783.51	3784.62	3786.52
(3,0,0)	3829.34	3883.62	3884.57	3884.99
(0,1,2)	3922.61	3996.74	4004.87	3991.34

2.3 SO_2

2.3.1 Potential Representation

In the case of SO_2 we also calculate the potential at the the B3LYP/def2-tzvp level of theory. The reference geometry corresponds to the equilibrium position, given in Cartesian coordinates in Table. S7.

Table S7 Cartesian coordinates of the equilibrium geometry of SO_2 in Ångström.

Atom	x	y	z
S	-0.003392	-0.005887	0.00
O	1.435506	0.001876	0.00
O	-0.705264	1.250360	0.00

The range of each normal coordinate along with the number of points (n_g) used to represent the potential are defined in Table. S8. Furthermore, one-dimensional cuts of the potential along each of the normal coordinates can be seen in Fig. S3.

Table S8 The range and number of grid points used along each normal coordinate for representing the SO_2 potential.

DOF	$q^{\min}$	$q^{\max}$	n_g
q_1	-80	80	41
q_2	-50	40	31
q_3	-40	40	31

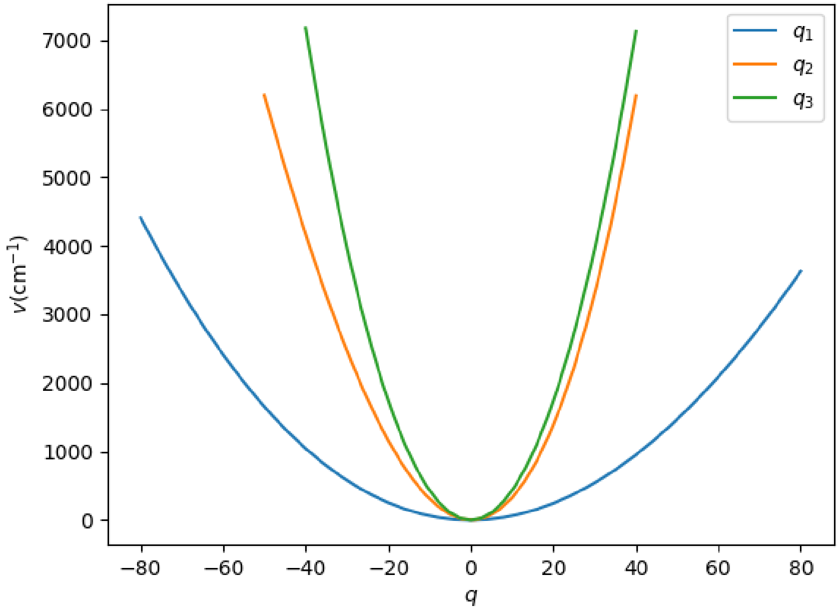

Figure S3 One-dimensional cuts of the SO_2 potential over the range of normal coordinates used. For each DOF, one coordinate is varied while the other two are fixed at zero.

2.3.2 Vibrational Transitions

Table. S9 shows the first 20 vibrational transitions energies and state assignments for SO_2. Again we see good agreement between all calculated transitions energies.

Table S9 Full table of the first 20 vibrational transition energies and assignments for SO_2. In the second column we show the experimental values, whereas columns 2-4 give the transitions calculated by CM-DVR, sine-DVR, and PS algorithm A1. Note all energies are given in cm^{-1}.

(v_1, v_2, v_3)	Exp.	CM-DVR	Sine-DVR	PS (A1))
(0,0,0)	0.00	0.00	0.00	0.00
(0,1,0)	517.69	517.45	517.48	517.48
(0,2,0)	1029.38	1034.62	1034.83	1034.60
(1,0,0)	1151.38	1164.62	1166.03	1165.17
(0,0,1)	1361.76	1357.94	1360.43	1359.53
(0,3,0)	1535.06	1552.04	1553.39	1552.72
(1,1,0)	1665.07	1679.27	1680.80	1679.87
(0,1,1)	1875.55	1870.89	1873.47	1872.55
(0,4,0)	2034.74	2072.29	2078.37	2068.94
(1,2,0)	2172.76	2193.73	2195.63	2194.22
(2,0,0)	2295.88	2327.51	2337.31	2320.76
(0,2,1)	2383.34	2383.58	2386.40	2385.23
(1,0,1)	2499.55	2511.57	2516.22	2514.56
(0,5,0)	2528.41	2602.81	2621.75	2612.47
(1,3,0)	2674.44	2708.87	2712.59	2703.50
(0,0,2)	2715.46	2718.19	2736.92	2710.13
(2,1,0)	2805.57	2839.56	2849.68	2832.70
(0,3,1)	2885.12	2896.58	2900.65	2899.02
(1,1,1)	3009.34	3021.72	3026.50	3024.81
(0,1,2)	3225.35	3156.27	3197.17	3122.92

3 Sobol Sampling

In sampling the configuration space for fitting potential energy surfaces (PESs) using Gaussian process regression (GPR), we utilise the quasi-random Sobol algorithm. This involves generating sequences of points, $\mathbf{x}_1, \ldots, \mathbf{x}_N$, in the d-dimensional unit cube $I^d = [0, 1]^d$ by minimising the discrepancy between points,

$$D^* (\mathbf{x}_1, \ldots, \mathbf{x}_N) = \sup_{\mathbf{x} \in I^d} |S_N (G_x) - N x_1 x_2 \ldots x_d|. \quad \text{(S20)}$$

Here $S_N(G_x)$ is the number of members $\{x_i\}$ in the subset G_x where,

$$G_x = [0, x_1) \times \ldots \times [0, x_d) \subset I^d. \quad \text{(S21)}$$

This allows for the generation of sequences that maximally span the space of the hyper-cube, while minimising the overlap of successively sampled points.

In this work we employ the implementation of Sobol sequence generation by Antonov and Saleev. This is based on choosing an primitive polynomial in $\mathbb{Z}_2$ for each dimension k,

$$x^{r_k} + a_{1,k}x^{r_k-1} + \ldots a_{r_k-1,j}x + 1, \tag{S22}$$

where $a_{ik} = 0$ or 1 for all i. With the coefficients in hand, the recurrence relation is defined,

$$m_{i,k} = 2a_{1,k}m_{i-1,k} \oplus 2^2 a_{2,k}m_{k-2,k} \oplus \ldots \oplus 2^{r-1}a_{r-1,k}m_{k-r+1,k} \oplus 2^r m_{k-r,k} \oplus m_{k-r,k}, \tag{S23}$$

for all $k > r_k$. Here, $\oplus$ defines the bitwise exclusive-or (XOR) operation. The initial values for the recurrence relation $m_{i,k}$ are freely chosen odd integers for all $i \leq r_k$, subject to the condition $m_{i,k} < 2^i$. From this, successive Sobol points are generated in each dimension k as,

$$x_{i,k} = x_{i-1,k} \oplus \nu_{i-1,k}, \tag{S24}$$

where $\nu_{i,k}$ are the so-called direction numbers,

$$\nu_{i,k} = \frac{m_{i,k}}{2^i}. \tag{S25}$$

Note that points are sampled following initialisation of the sequence in Eq. S24 in each dimension as $x_{0,k} = 0$.

3.1 Effect of Sampling on Molecular Calculations

As discussed in the main manuscript, the calculated vibrational transitions of both SO_2 and NO_2 are accurately recreated from a PES fit from 128 Sobol sampled points using Gaussian Process Regression (GPR). In Fig. S4, we show the average difference of vibrational

transitions calculated using CM-DVR (top panel) and sine-DVR (bottom panel) relative to PS predicted transitions, where the PS transitions are calculated using a PES constructed from 64 Sobol points. Here, the predictions for NO_2 deviate by around 10-20 cm^{-1}. Whereas, one is still able to achieve sub-wavenumber accuracy for SO_2 relative to CM-DVR, where eight of nine transitions are within ± 1 cm^{-1}. In addition, we see that eight of nine transitions are within ± 2.5 cm^{-1} of the sine-DVR predictions.

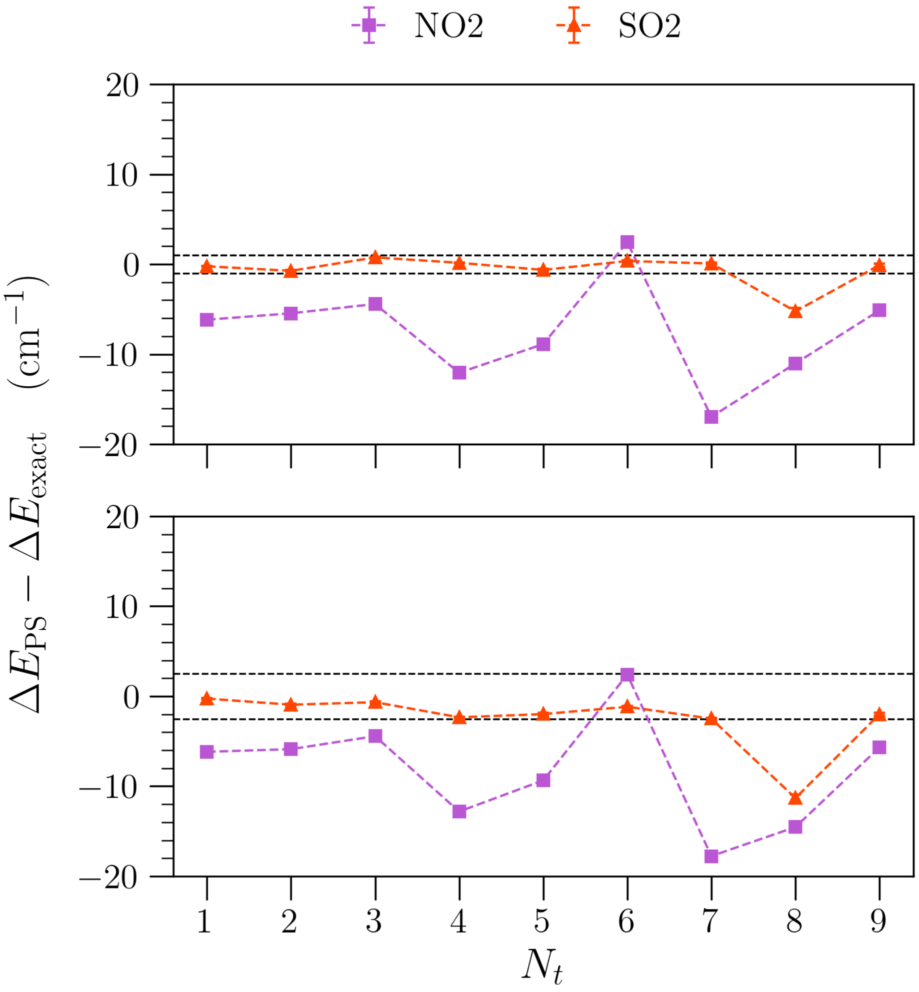

Figure S4 Average differences between PS predicted transition energies calculated using a GPR-PES (fit using 64 Sobol points), and transition energies predicted by CM-DVR (top) and sine-DVR (bottom) using pure DFT potentials. Note, dashed black lines mark transitions within ± 1 cm^{-1} and ± 2.5 cm^{-1} in the top and bottom panels respectively.

4 PS Function Library

Table S10 Table of input functions that initialise the workspace matrix $\mathbf{M}$

Function ID	Operation	Elements
1	$M_{ij} \to 1$	$\forall i = j$
2	$M_{ij} \to 1$	$\forall i, j$
3	$M_{ij} \to 1$	$\forall i \neq j$
4	$M_{ij} \to 1$	$\forall j \in [i, i \pm 1]$

Table S11 Table of internal functions that operate on the workspace matrix $\mathbf{M}$. Here functions that involve the same operators are grouped together, thus rows may correspond to multiple instances of an internal function with a different argument (a). In addition, the fourth column indicates if the function is applied to all elements of $\mathbf{M}$, or only the off-diagonal terms. Note, in the row marked ‡, the 13 functions represented by f are defined in Table. S12

Function IDs	Function (f)	Argument (a)	Elements
1-6	$M_{ij} \to M_{ij} + a$	$[\pi, 2, 3, 4, m, L]$	$\forall i = j$
7-12	$M_{ij} \to M_{ij} - a$	$[\pi, 2, 3, 4, m, L]$	$\forall i = j$
13-18	$M_{ij} \to M_{ij}/a$	$[\pi, 2, 3, 4, dx^2, m, L]$	$\forall i = j$
19-25	$M_{ij} \to M_{ij} \times a$	$[-1, \pi, 2, 3, 4, dx^2, m, L]$	$\forall i = j$
26-31	$M_{ij} \to M_{ij} + a$	$[\pi, 2, 3, 4, m, L]$	$\forall i \neq j$
32-37	$M_{ij} \to M_{ij} - a$	$[\pi, 2, 3, 4, m, L]$	$\forall i \neq j$
38-44	$M_{ij} \to M_{ij}/a$	$[\pi, 2, 3, 4, dx^2, m, L]$	$\forall i \neq j$
45-51	$M_{ij} \to M_{ij} \times a$	$[\pi, 2, 3, 4, dx^2, m, L]$	$\forall i \neq j$
52-54	$M_{ij} \to M_{ij} \times e^{a(x_i - x_j)^2}$	$[-1, -0.5, 1]$	$\forall i \neq j$
55-67 ‡	$M_{ij} \to f(M_{ij})$	-	$\forall i = j$
68-71	$M_{ij} \to M_{ij}^a$	$[0.5, 2, 3, 4]$	$\forall i = j$
72-74	$M_{ij} \to M_{ij} \times \lvert x_i - x_j \rvert^a$	$[-1, -2, -3]$	$\forall i \neq j$
75-78	$M_{ij} \to M_{ij} \times (x_i - x_j)^a$	$[-4, -2, 2, 4]$	$\forall i \neq j$
79	$M_{ij} \to M_{ij} \times e^{(x_i - x_j)^a}$	$[-2]$	$\forall i \neq j$
80	$M_{ij} \to M_{ij} \times (-1)^{i-j}$	-	$\forall i \neq j$

Table S12 The function applied to M_{ij} as represented by f in the row marked ‡ of Table. S11. Here I_0 refers to the modified Bessel function of the first kind with order zero.

Function ID	Function
55	sin
56	cos
57	sinc
58	cosh
59	sinh
60	tanh
61	exp
62	$2^{M_{ij}}$
63	$\exp(M_{ij}) - 1$
64	log
65	$\log_{10}$
66	$\log(1 + M_{ij})$
67	I_0

5 PS Algorithms

The set of seven PS algorithms that converge in all tests, and that predict reliable transition energies for all three tri-atomic molecules are listed below. Note, we only list the KE matrix elements as the potential is always subsequently added to the diagonal of this to yield the total Hamiltonian. In addition, all algorithms have a tri-diagonal matrix structure.

5.1 Pseudo-Variationally Trained

A1:

$$T_{jk} = \begin{cases} \frac{1}{L^9 dx^2} & j = k \\ -\frac{m}{2dx^2} & j = k \pm 1 \end{cases} \tag{S26}$$

A2:

$$T_{jk} = \begin{cases} \tanh\left(m\left(1 - L\right)\right) & j = k \\ -\tanh\left(\frac{Lm - m}{2dx^2}\right) & j = k \pm 1 \end{cases} \tag{S27}$$

A3:

$$T_{jk} = \begin{cases} \log_{10}\left(\mathrm{sinc}\left(1\right)\right) & j = k \\ \frac{1}{2dx^2}\log_{10}\left(\frac{\mathrm{sinc}(1/L)}{3\pi}\right) & j = k \pm 1 \end{cases} \tag{S28}$$

A4:

$$T_{jk} = \begin{cases} -\frac{1}{2}m + I_0\left(\frac{\pi\mathrm{sinc}(1)}{4}\right) & j = k \\ \frac{2}{dx^2}\left(-\frac{1}{2}m + I_0\left(\frac{\pi\mathrm{sinc}(1)}{4}\right)\right) & j = k \pm 1 \end{cases} \tag{S29}$$

A5:

$$T_{jk} = \begin{cases} \frac{2}{dx^2} & j = k \\ \frac{(-1)^{j-k}m^2}{2dx^2} & j = k \pm 1 \end{cases} \tag{S30}$$

5.2 Target Function Trained

A6:

$$T_{jk} = \begin{cases} -\frac{3\cos(1)-3+m}{2} & j = k \\ -\frac{1}{2dx^2}\left(\frac{3\cos(1)-3}{L} + m\right) & j = k \pm 1 \end{cases} \tag{S31}$$

A7:

$$T_{jk} = \begin{cases} \frac{1}{3}\sinh\left(1\right) - m & j = k \\ \frac{1}{2dx^2}\left(\frac{1}{3mL}\sinh\left(1\right) + \frac{1}{L} - m\right) & j = k \pm 1 \end{cases} \tag{S32}$$
